# Evolutionary-driven *C-MYC* gene expression in mammalian fibroblasts

**DOI:** 10.1038/s41598-020-67391-x

**Published:** 2020-07-06

**Authors:** Marcelo T. Moura, Roberta L. O. Silva, Ludymila F. Cantanhêde, José C. Ferreira-Silva, Pábola S. Nascimento, Ana M. Benko-Iseppon, Marcos A. L. Oliveira

**Affiliations:** 1 Department of Veterinary Medicine, Federal Rural University of Pernambuco-UFRPE, Recife, Brazil; 2 Department of Genetics, Federal University of Pernambuco-UFPE, Recife, Brazil

**Keywords:** Evolutionary developmental biology, Reprogramming, Biotechnology, Cancer, Cell biology, Developmental biology, Evolution, Genetics, Molecular biology, Stem cells

## Abstract

The extent to which mammalian cells share similar transcriptomes remains unclear. Notwithstanding, such cross-species gene expression inquiries have been scarce for defined cell types and most lack the dissection of gene regulatory landscapes. Therefore, the work was aimed to determine *C-MYC* relative expression across mammalian fibroblasts (*Ovis aries* and *Bos taurus*) via cross-species RT-qPCR and comprehensively explore its regulatory landscape by in silico tools. The prediction of transcription factor binding sites in *C-MYC* and its 2.5 kb upstream sequence revealed substantial variation, thus indicating evolutionary-driven re-wiring of cis-regulatory elements. *C-MYC* and its downstream target *TBX3* were up-regulated in *Bos taurus* fibroblasts. The relative expression of *C-MYC* regulators [*RONIN* (also known as *THAP11*), *RXRβ*, and *TCF3*] and the *C-MYC*-associated transcript elongation factor *CDK9* did not differ between species. Additional in silico analyses suggested *Bos taurus*-specific *C-MYC* exonization, alternative splicing, and binding sites for non-coding RNAs. *C-MYC* protein orthologs were highly conserved, while variation was in the transactivation domain and the leucine zipper motif. Altogether, mammalian fibroblasts display evolutionary-driven *C-MYC* relative expression that should be instructive for understanding cellular physiology, cellular reprogramming, and *C-MYC*-related diseases.

## Introduction

Cell types arise by stepwise acquisition of specific gene expression programs during development and have stable cellular phenotypes throughout organism adulthood^1^. The transcriptome of any given cell type is the culmination of contextual interplay between intrinsic factors (e.g., transcription factors—TFs, non-coding RNAs) and extrinsic factors from the extracellular milieu under the context of species-specific variation that may have arisen during evolutionary trajectories^2–6^.

Several mammalian species have similar transcriptomes across multiple organs^7–9^, thus suggesting that few loci account for species-specific organ function and response to environmental cues or disease states^10^. Hence, cross-species gene expression analysis becomes an attractive strategy to assist unraveling evolutionary-driven differences in the gene expression regulatory landscape^8^. Although this comparative transcriptomic approach—largely based upon RNA sequencing—has been informative, most of them account for analysis carried out with whole organs^7,9^. Therefore, cell-type specific interrogations and cross-species comparisons of relative gene expression have remained mostly unexplored.

Unbiased cross-species gene expression analysis may pinpoint differences in species-specific relative gene expression at a given locus^10–12^. Recently, one such report has found that the expression of two TFs (*TLR4* and *ZFX*) were up-regulated in *Bos taurus* (*B. taurus*) fibroblasts compared to *Ovis aries* (*O. aries*) counterparts using rigorous reverse transcription quantitative PCR (RT-qPCR) normalization^12^. The dissection of such differences between closely related species may be fruitful due to the expected similarities in their gene expression regulatory landscapes^11,12^.

Species-specific differences in gene expression suggest the rewiring of gene regulatory landscapes. The evolutionary-driven divergences in such networks are byproducts of cis-regulatory elements (CRE) turnover^13,14^ and sequence variation in TFs^15^. The CREs are DNA sequences that contribute to the regulation of gene expression, usually containing several TF-binding sites (TFBS) and may include sequence elements that affect mRNA stability and translation^15^. The CRE evolution is the result of transposable elements acquiring new regulatory roles or sequence evolution within chromatin regions with regulatory potential^13,14^. TFs are also source of novel gene regulatory landscapes, thus resulting in TF orthologs with different functions or target genes^13^. The interplay between TF and CRE variation may limit the conservation of TFBS^4,16,17^. For instance, *OCT4* (also known as *POU5F1*) and *NANOG* share few (~ 5%) homologous TFBS in mouse and human embryonic stem cells^17^. Therefore, integrative analysis coupling gene expression analysis and TFBS mapping should contribute toward elucidating evolutionary-driven divergences in gene regulatory landscapes.

*C-MYC* is an interesting TF to investigate species-specific gene expression patterns because of its contribution to several cellular processes. *C-MYC* forms a unique transcriptional network in pluripotent cells^18,19^, regulates over one-tenth of the transcriptome^20,21^, elicits chromatin architecture modulation^22^, participates in DNA replication and cell cycle control^23,24^, modulates apoptosis^25^, and may increase overall transcription in a given cell^26^. In the context of cellular reprogramming, *C-MYC* enhances the conversion of somatic cells into induced pluripotent stem (iPS) cells and accelerates this process in both human and mouse systems^19,27^.

*C-MYC* levels have profound effects in cellular physiology^28,29^. Therefore, this locus is regulated by autoregulation^30^ and several other factors (*RONIN* also known as *THAP11*, *RXRβ*, *TCF3*, *ZFX,* among others) at the transcription, post-transcription, and post-translation levels^28–37^. The analysis of regulatory sequences may identify potential sources of species-specific *C-MYC* relative expression. The aim of this work was to determine the relative expression of *C-MYC, CDK9,* and *C-MYC* regulators between *O. aries* and *B. taurus* fibroblasts. In silico tools were further applied to identify potential sources of *C-MYC* relative expression between species at the mRNA, protein, and CRE levels.

## Results

### Analysis of *C-MYC* cis-regulatory elements in *Ovis aries* and *Bos taurus* genomes

**Figure 1:**
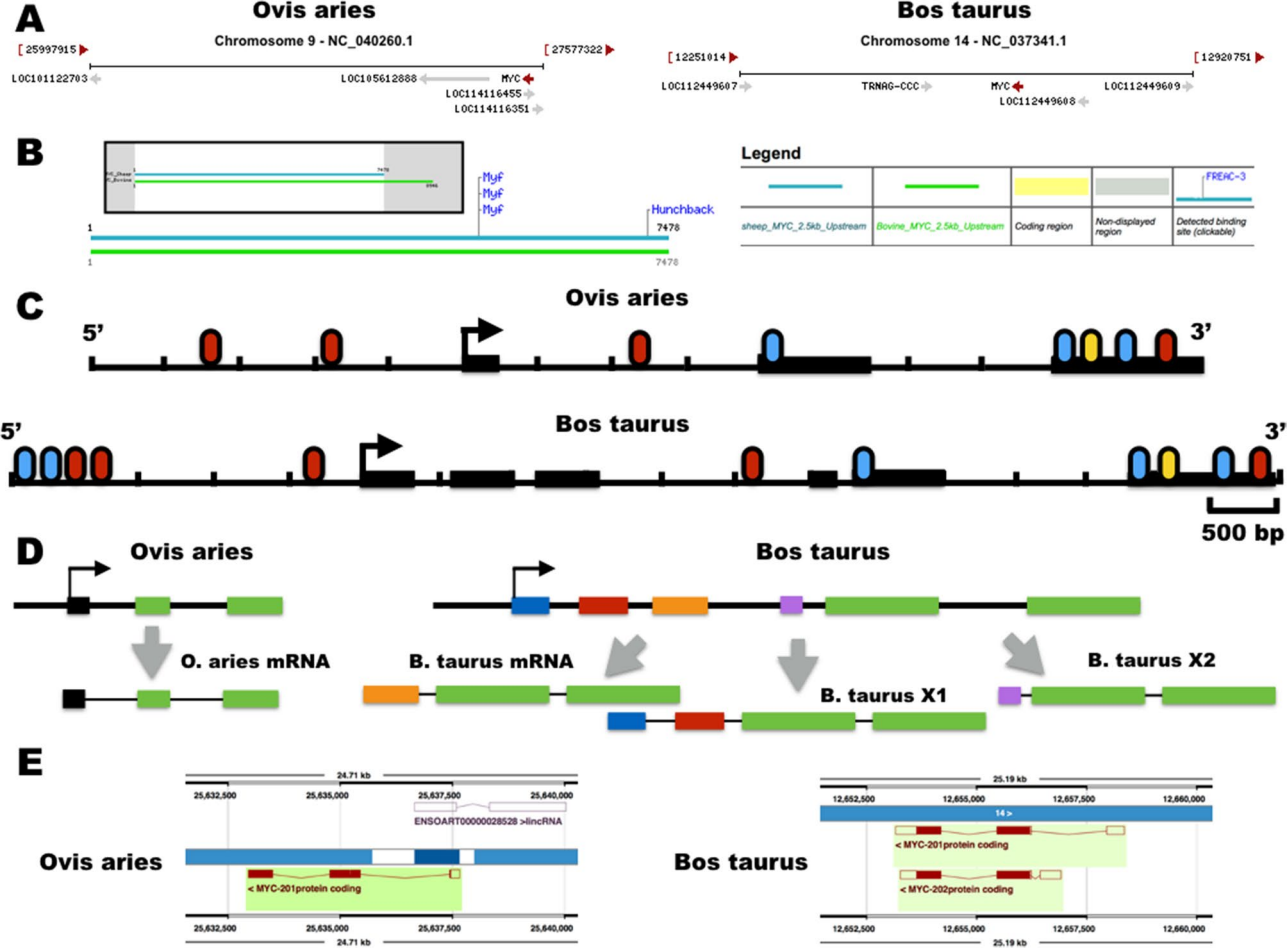
Cross-species analysis of the *C-MYC* locus. (**A**) Genomic context of *O. aries* (reference genome oar_rambouillet_v1.0) and *B. taurus* (reference genome ARS_UCD1.2) *C-MYC* gene orthologs retrieved from genome data viewer (NCBI). (**B**) Prediction of conserved transcription factor binding sites (TFBS) between *O. aries* (blue lines) and *B. taurus* (green lines) using ConSITE in the *C-MYC* locus and 2.5 kb upstream sequence of the transcription start site (TSS). The gray boxes show the non-conserved DNA sequences between *C-MYC* loci. (**C**) Representation of TFBS for *C-MYC* (yellow), *RXRβ* (blue), and *TCF3* (red) in the *C-MYC* locus and 2.5 kb upstream sequence of the TSS. Exons were outlined as black boxes and the TSS was indicated with an arrow. (**D**) Schematic representation of predicted alternative splicing in the *B. taurus C-MYC* locus by analysis of reference mRNA sequences. (**E**) Prediction of alternative splicing in the *B. taurus C-MYC* locus in the Ensembl genome browser.

The *C-MYC* locus is located on the *O. aries* chromosome 9 (genome version oar_rambouillet_v1.0) and on chromosome 14 of the *B. taurus* genome (genome version ARS_UCD1.2). The *C-MYC* gene is transcribed by the minus strand of the DNA in both *O. aries* and *B. taurus* (see Fig. 1A)*.* The *B. taurus C-MYC* locus has six exons and O. aries has three (as found in the mouse and humans). An unbiased prediction of conserved TF binding sites (TFBS) at the *C-MYC* locus and 2.5 kb upstream the transcription start site between *O. aries* and *B. taurus* retrieved one site for *Hunchback* and three overlapping sites for *MYF* (see Fig. 1B; Supplementary Fig. S1). Since Hunchback is a *Drosophila melanogaster*-specific TF, only *MYF* TFBS are potentially conserved between *O. aries* and *B. taurus* (see Fig. 1B). The TFBS prediction for *C-MYC*, *RXRβ*, and *TCF3* demonstrated species-specific variation (see Fig. 1C; Supplementary Fig. S2), thus reinforcing the notion of evolutionary-driven re-wiring of CREs. The current genome assemblies suggest different number of *C-MYC* mRNA isoforms between species, thus implying *B. taurus*-specific alternative splicing (see Fig. 1D). The Ensembl genome browser displayed two *C-MYC* mRNA isoforms in the current *B. taurus* genome assembly ARS_UCD1.2 and one *C-MYC* isoform in *O. aries* (see Fig. 1E), although based on a previous sheep genome version (oar_v3.1).

### Relative expression of *C-MYC*, *CDK9*, and *C-MYC*-regulators in *Ovis aries* and *Bos taurus* fibroblasts

Primer efficiency was determined using *O. aries* and *B. taurus* fibroblast cDNA (see Table 1). All primers reached qPCR efficiency threshold, which fluctuated from 94.52 to 109.33%. The correlation coefficient of such qPCR reactions varied from − 0.990 to − 0.998, while their respective slopes ranged from − 3.12 to − 3.46 and Y intercepts from 32.49 to 38.40 (see Table 1). Hence, the RT-qPCR normalization relied on a set of previously validated reference genes (RGs) using five software (GeNorm, Normfinder, BestKeeper, Delta CT method, and RefFinder) under identical experimental conditions^12^.

**Table 1:** Primer efficiency (E), coefficient correlation (R), slope, and Y intercept derived from the standard curve of each transcript in the study via RT-qPCR.

Gene symbol	cDNA	E (%)	Correlation coefficient (R)	Slope	Y intercept
*CDK9*	Bulk	97.97	− 0.997	− 3.37	32.08
*CMYC*	Bulk	100.28	− 0.993	− 3.32	31.28
*RONIN (THAP11)*	*Ovis aries*	97.49	− 0.997	− 3.38	34.28
*RONIN (THAP11)*	*Bos taurus*	109.33	− 0.995	− 3.12	32.49
*RXRβ*	Bulk	106.93	− 0.993	− 3.17	33.62
*TBX3*	Bulk	98.33	− 0.990	− 3.36	38.40
*TCF3*	Bulk	94.52	− 0.998	− 3.46	34.77

Bulk cDNA: 1:1 mixture of *Ovis aries* and *Bos taurus* cDNA.

**Figure 2:**
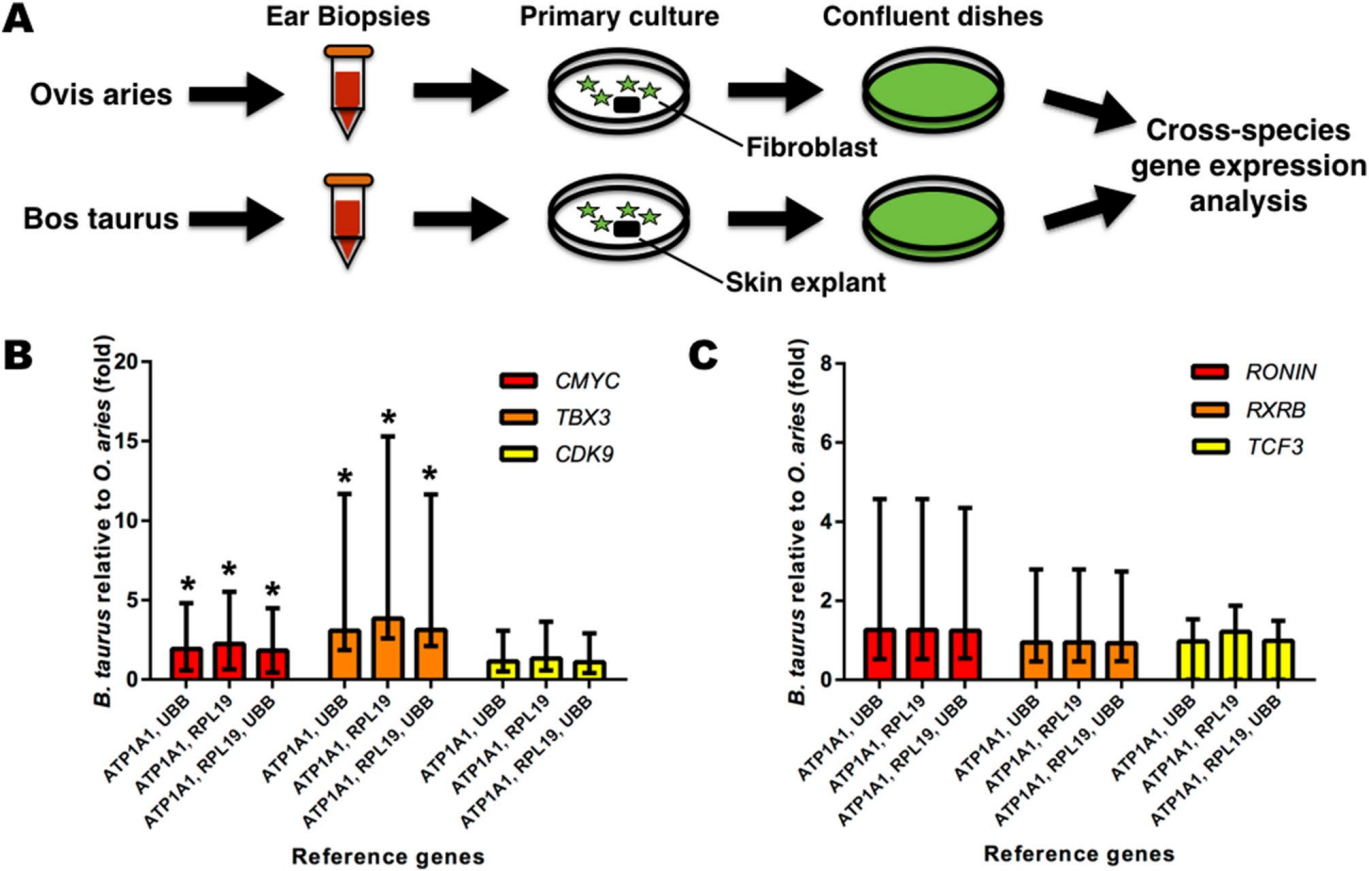
Relative expression of *C-MYC, CDK9*, and *C-MYC* regulators in mammalian fibroblasts. (**A**) Experimental design for cross-species gene expression analysis. (**B**) Relative expression of *CDK9* (Yellow), *C-MYC* (red), and *TBX3* (Orange). (**C**) Relative expression of *RONIN* (red), *RXRβ* (Orange), and T*CF3* (Yellow). The relative expression was determined as expression fold (x) of the relative expression of the *B. taurus* relative to *O. aries* ortholog. Standard error range (+ /−) was calculated by REST^52^.

The RT-qPCR assay determined the *C-MYC* relative expression in unmodified mammalian fibroblasts (see Fig. 2A). *C-MYC* relative expression was 1.82–2.23 fold higher in *B. taurus* in comparison to *O. aries* (P < 0.001; see Fig. 2B). Further, the *C-MYC* downstream target *TBX3* was up-regulated in *B. taurus* by 3.07–3.84-fold (P < 0.001). In spite of different RGs, *C-MYC* and *TBX3* relative expression differed between species (see Fig. 2B).

To infer if mammalian fibroblasts may have variable global transcription output, the RT-qPCR determined the relative expression of the transcript elongation factor (*CDK9*). However, *CDK9* relative expression was similar between species (P = 0.64; see Fig. 2B). To explore potential sources of *C-MYC* relative expression between species, gene expression analysis measured the relative expression of *C-MYC* trans-acting regulators. The relative expression of the *C-MYC* negative regulators did not differ between *O. aries* and *B. taurus* fibroblasts (*RONIN:* P = 0.40; *RXRβ:* P = 0.75; and *TCF3:* P = 0.43; see Fig. 2C). Moreover, alternative RGs did not affect the results of the gene expression analysis (see Fig. 2C).

### Analysis of *C-MYC* mRNA regulatory sequences and N^6^-methyladenosine in *Ovis aries *and *Bos taurus* orthologs

**Figure 3:**
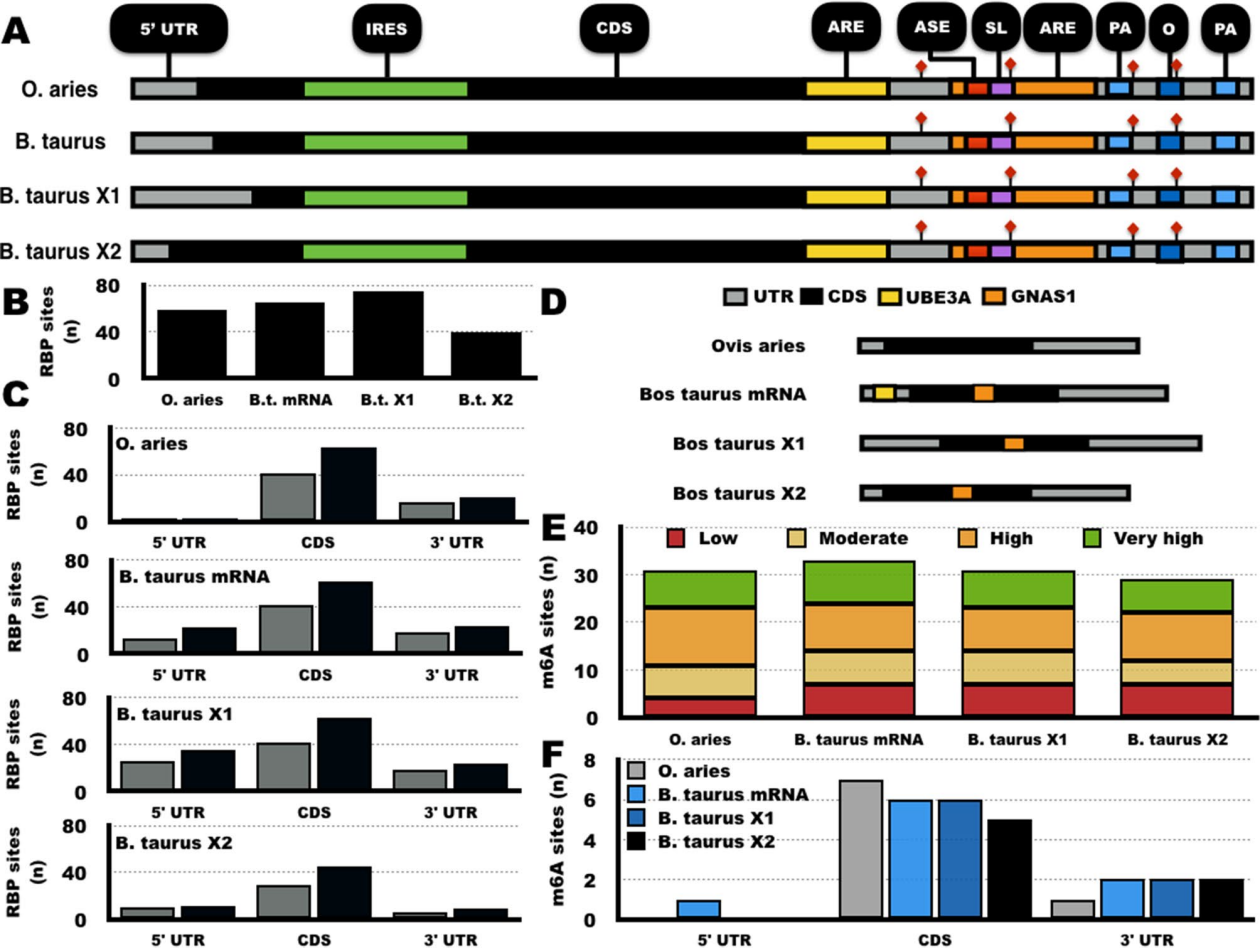
In silico analysis of *C-MYC* mRNA orthologs. (**A**) Representation of *O. aries* and *B. taurus C-MYC* mRNA isoforms highlighting the regulatory sequences*.* (**B**) Total number of predicted RNA-binding proteins (RBPs) in *C-MYC* mRNA isoforms. (**C**) Distribution of predicted RBPs in *C-MYC* mRNA isoforms. (**D**) Schematic representation (5ʹ to 3ʹ direction) of predicted binding sites for the non-coding RNAs *GNAS1* (Orange) and *UBE3A* (Yellow). (**E**) Total prediction of N^6^-methyladenosine (m^6^A) sites. (**F**) m^6^A sites with very high predictive scores and their distribution relative to the coding sequence (CDS). ARE: AU-rich elements. ASE: AU-rich sequence element UUUN [A/U] U. IRES: internal ribosome entry site. O: 3ʹ UTR octamer. PA: polyadenylation site. Red diamonds: AUUUA motifs. SL: Stem loop/AU-rich sequence. UTR: untranslated region.

The *C-MYC* locus is under rigorous transcriptional and post-transcriptional regulation. The analysis of mRNA sequence conservation between *C-MYC* orthologs may point out sources of variable relative expression between species. Based on current genome assemblies, the *O. aries* has one reference mRNA sequence while *B. taurus* has three isoforms (see Supplementary Fig. S3). The annotation of characterized *C-MYC* mRNA regulatory sequences across mammals supports high sequence conservation between *O. aries* and *B. taurus* (see Fig. 3A). The 5ʹ untranslated region (UTR) of both *O. aries* and *B. taurus* orthologs display most of the sequence variation between these species (see Supplementary Fig. S3). In contrast, coding sequences (CDS) displayed high sequence conservation, including the coding region instability determinant (CRD) in the last 249 nucleotides of the CDS, adjacent to the ribosomal pausing site. The 3ʹ UTR sequence of the four *O. aries* and *B. taurus C-MYC* orthologs also have high sequence conservation (see Supplementary Fig. S4) and share multiple regulatory sequences (see Fig. 3A), such as AU-rich octamer, AU-rich stem loop, AU-rich elements, five-nucleotide motifs, polyadenylation sites, and the AU-rich sequence element (see Supplementary Fig. S4).

Several reports showed that RNA-binding proteins (RBPs) contribute to *C-MYC* mRNA stability. The prediction of *C-MYC*-binding RBPs showed that *C-MYC* mRNAs have multiple putative binding sites (see Fig. 3B). *C-MYC* mRNA orthologs showed similar number of binding sites for RBPs, albeit more frequent in the CDS (see Fig. 3C). The functional annotation of predicted RBPs most covered by TFs (12%), although few of these TFs were characterized to bind RNA. Further, mRNA motif predictions suggest *B. taurus*-specific binding sites for the non-coding RNAs *GNAS1* and *UBE3A* (see Fig. 3D).

The N^6^-methyladenosine (m^6^A) is an epigenetic modification of RNA that has important regulatory roles. The M^6^A prediction was also investigated in *O. aries* and *B. taurus C-MYC* mRNA orthologs. The *O. aries C-MYC* mRNA displayed 31 potential m^6^A sites, while *B. taurus C-MYC* mRNA isoforms had 29 to 33 m^6^A potential sites (see Fig. 3E). Further, both *O. aries* and *B. taurus C-MYC* mRNA did not display differences in m^6^A sites according to very high (P = 0.51), high (P = 0.78), moderate (P = 0.22) or low (P = 0.97) predictive scores (see material and methods; see Fig. 3E). A schematic representation demonstrates that highly predicted m^6^A sites were mostly found in the CDS and similarly distributed among *O. aries* and *B. taurus C-MYC* mRNAs (see Fig. 3F).

### Comparative analysis of *Ovis aries* and *Bos taurus C-MYC* protein sequences and potential sites for post-translational modification

**Figure 4:**
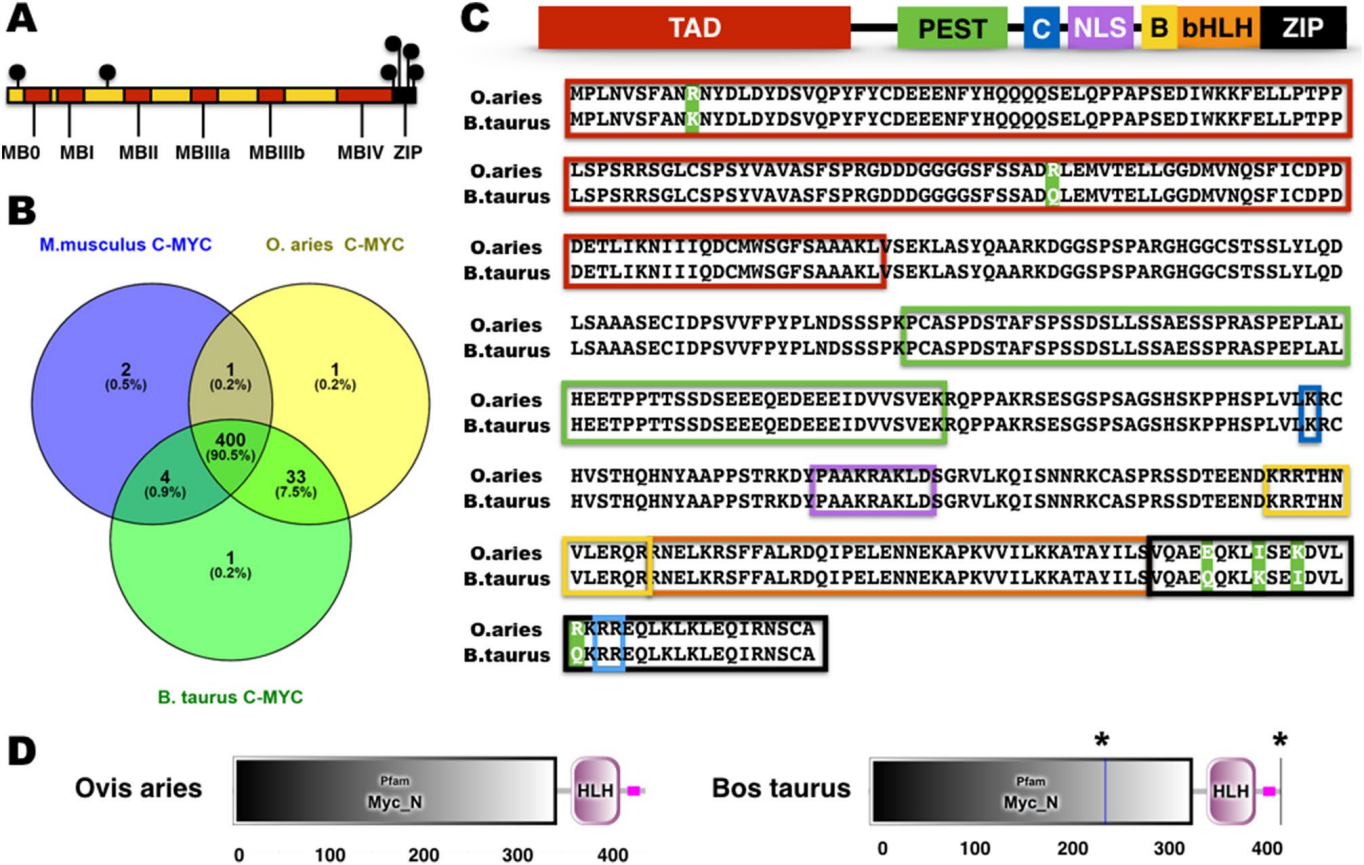
In silico analysis of *C-MYC* protein orthologs. (**A**) Representation of *C-MYC* of both *O. aries* and *B. taurus C-MYC* orthologs and their non-conserved residues (black spheres)*.* (**B**) *C-MYC* boxes (I–IV) outlined in red. Conservation of *C-MYC* protein among selected mammalian species. (**C**) Motif analysis between *O. aries* and *B. taurus C-MYC* orthologs. (**D**) Secondary structure prediction of *O. aries* and *B. taurus C-MYC* orthologs describing the transactivation domain (TAD; gray boxes), the basic helix-loop-helix DNA-binding domains (bHLH; large pink boxes), and low complexity region (small pink boxes), and intron sites (*). B: basic region. C: calpain cleavage site. NLS: nuclear localization signal. PEST: motif rich in proline [P], glutamic acid [E], serine [S], and threonine [T]. ZIP: leucine zipper motif. Light blue box: Conserved amino acid residues (RR) required for *C-MYC/MAX* heterodimer formation.

*C-MYC* is under complex regulation by post-translational modification (PTM). The alignment of *O. aries* and *B. taurus C-MYC* protein orthologs showed a sequence conservation of 98.63% (433/439 residues; see Supplementary Fig. S5). Further, distinct amino acid residues between *O. aries* and *B. taurus* species were found in the transactivation domain (TAD; N-terminus) but enriched in the ‘leucine zipper’ motif in the C-terminus (see Fig. 4A). In comparison to the *Mus musculus* (mouse) *C-MYC* protein, *O. aries* and *B. taurus* proteins share 91.14% (413/440 residues) and 90.45% (398/440 residues) homologies, respectively (see Fig. 4B; see Supplementary Fig. S5). *C-MYC* domains showed high conservation between *O. aries* and *B. taurus* (see Fig. 4C). The calpain cleavage site and the bHLH DNA-binding domain were identical among species, thus leading to overlapping E-BOX DNA binding motifs. The motif rich in proline [P], glutamic acid [E], serine [S], and threonine [T] (PEST) domain and the nuclear localization signal were also identical between *O. aries* and *B. taurus* (see Fig. 4C). Further, *C-MYC* orthologs display substitutions in only two residues in the TAD (i.e., within *C-MYC* degron site and the auto-repression sequences). The sequence variation in *C-MYC* protein orthologs did not affect the secondary structure prediction of key domains (see Fig. 4D). Hence, the fourth leucine of this leucine-rich motif was not found in both *O. aries* and *B. taurus,* although it was not within the amino acid residues expected for the *C-MYC/MAX* heterodimer formation (see Fig. 4C)*.*

Most PTM sites in the *Homo sapiens* (human) *C-MYC* protein were conserved residues in *O. aries* and *B. taurus* orthologs (see Supplementary Fig. S6). The exceptions to this rule are three substitutions of threonine to alanine (i.e., residues 8, 78, and 343), which could be (albeit uncharacterized) phosphorylation sites (see Supplementary Fig. S6). Nonetheless, no predicted PTM differed between *O. aries* and *B. taurus* orthologs.

**Figure 5:**
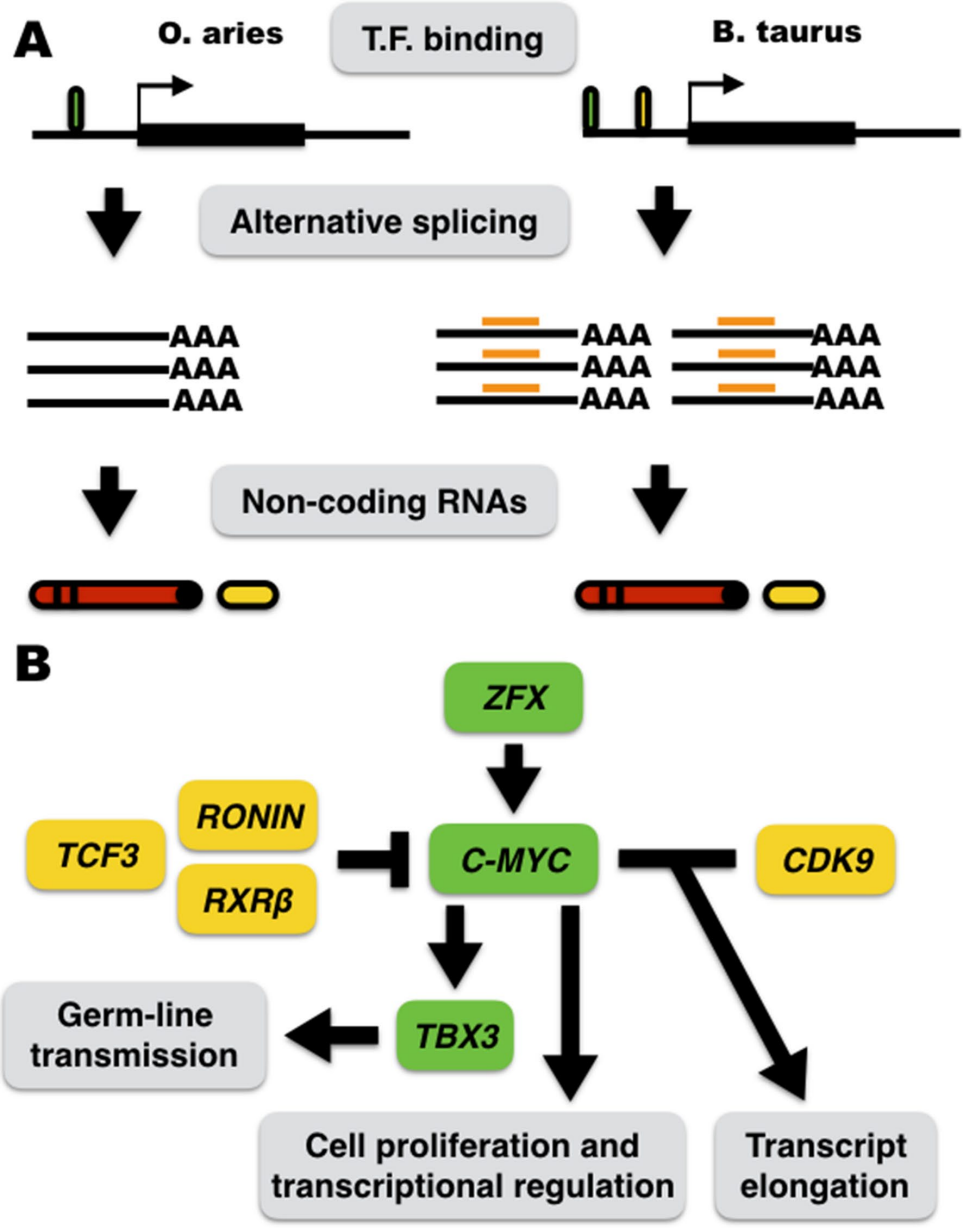
Scheme highlighting potential sources of evolutionary-driven *C-MYC* relative expression in mammalian fibroblasts. (**A**) Representation of the *C-MYC* locus in *O. aries* and *B. taurus* highlighting potential evolutionary-driven divergences in their regulatory landscape. (**B**) *C-MYC* gene transcription-based circuitry investigated in this study. *C-MYC* repressors and activators outlined by “T” and “arrows”, respectively. Non-significant cross-species gene expression was described in yellow and *B. taurus* upregulated genes in green. Potential cellular effects of *B. taurus*-specific *C-MYC* and *TBX3* upregulation highlighted in gray boxes. Moura et al*.* described the data on *ZFX* in a recent report^12^. TF: transcription factor.

Based upon the results above, a scheme was described with potential evolutionary-driven differences between *O. aries* and *B. taurus C-MYC* gene orthologs (see Fig. 5A). An outline was also presented describing the relative expression of *C-MYC*, *CDK9*, and *C-MYC*-regulators between *B. taurus* and *O. aries*, their interactions to *C-MYC* transcription, and potential cellular effects that may arise from such species-specific gene expression signatures (see Fig. 5B)*.*

## Discussion

The prediction of TFBS at the *C-MYC* locus for its encoded protein (autoregulation) and some of its negative regulators revealed substantial variation in TFBS numbers and locations. Genome-wide analyses have demonstrated that TFBS were poorly conserved across mammalian genomes^4,38,39^, perhaps due to CRE re-wiring caused by transposable elements^17,38–40^. In turn, relative expression of known *C-MYC*-negative regulators *RONIN*, *RXRβ*, and *TCF3*^31–33^ was similar between *O. aries* and *B. taurus*. This fact suggests that expression levels of these TFs may not affect species-specific gene expression patterns in mammalian fibroblasts. A next logical step is to investigate the conservation of TFBS for *ZFX* at *O. aries* and *B. taurus C-MYC* locus and its impact on *TBX3* gene expression, due to their connection to this proto-oncogene^41,42^. The re-wiring of TF networks must be a significant driving force in the regulatory modes of the *C-MYC* locus across species and variation at CREs should explain some (if not most) of the gene expression variation due to the evolutionary trajectories.

*Bos taurus* fibroblasts express *C-MYC* to a greater extent than *O. aries*. The species-specific *C-MYC* relative expression motivated the exploration of evolutionary-driven divergences in the regulatory landscape at the genomic, mRNA and protein levels. The *B. taurus C-MYC* locus contains six exons, in contrast to the common three-exon structure found in *Ovis aries* and other mammals. Further, *B. taurus C-MYC* locus is expected to carry out alternative splicing, in contrast to *O. aries.* The creation of new exons and emergence of alternative splicing are clear indications of locus evolution, since these processes tend to avoid negative pleiotropy of evolving TFs^15^. In silico analyses suggested more sequence variation in *C-MYC* mRNA orthologs. Rather remarkably, most mRNA sequence variation was found in the 5ʹ UTR of *O. aries* and *B. taurus C-MYC* mRNA, although its significance remains elusive. In other species, the *C-MYC* 5′ UTR was found to contain an internal ribosome entry site^43^, to contribute to cancer-associated cellular phenotypes^44^ and to translational efficiency^45^. Binding sites for non-coding RNAs were another potential source of regulatory variation between *C-MYC* mRNA orthologs. There is extensive evidence of long non-coding RNAs and microRNAs regulatory hubs at the *C-MYC* locus in both mice and humans^36,37^. The evidence of non-coding RNAs as potential regulators of the *C-MYC locus* in *O. aries* and *B. taurus* remains preliminary due to the limited availability of in silico tools for these species and should be focus of further work. Nonetheless, cross-species gene expression analysis coupled with bioinformatics identified a small subset of CREs that may explain species-specific *C-MYC* relative expression. Future research should focus on interrogating the role of each CRE on *C-MYC* transcription across mammals. A detailed characterization of *C-MYC* enhancers in *O. aries* and *B. taurus* may be another fruitful endeavor because these CREs evolve more rapidly than promoters and gene expression^14,46^.

The higher *C-MYC* relative expression in *B. taurus* than *O. aries* may reflect greater differences in the transcriptome of fibroblasts from these species. *C-MYC* interacts with *CDK9* from the elongation complex *P-TEFb* (formed by *CDK9* and cyclins T1 and T2) and releases hundreds of transcripts from RNA polymerase II-mediated transcriptional elongation pausing^26^. However, *CDK9* transcript abundance was similar between *O. aries* and *B. taurus*. The pharmacological *CDK9* down-regulation may lead to increased *C-MYC* expression in human cancer cells^47,48^, although the results described here do not accommodate such compensatory mode. Gene expression analysis using identical cell numbers or single-cell analysis coupled with pharmacological *CDK9* modulation should resolve such discrepancies. Nonetheless, the results showed that *C-MYC* had variable gene expression levels across mammalian fibroblasts and this difference may affect the transcriptome of such cells.

These facts on *C-MYC* species-specific gene expression are paramount for understanding biological processes at an evolutionary level, for modeling human conditions in animal models, and improved understanding of cellular reprogramming. *C-MYC* acts pleiotropically in mammalian cells, particularly as a pro-survival factor and inducing cell proliferation^23,24^. For instance, *C-MYC* overexpression increases the efficiency of cellular reprogramming in mice and humans^19,27^. Higher endogenous *C-MYC* and *TBX3* expression or their overexpression during reprogramming was associated with germ-line contribution of iPS cell lines^19,27,41^. It would be informative to determine if species-specific *C-MYC* levels correlate with iPS reprogramming efficiency and kinetics. Alternatively, the cross-species analysis of *C-MYC* could guide the development of improved animal models of *C-MYC*-driven cancer, such as mice expressing human *C-MYC* protein^49^, by focusing on adjusting oncogenic *C-MYC* expression between mammalian models and patient samples.

In conclusion, mammalian fibroblasts display evolutionary-driven *C-MYC* relative expression, most probably due to rewiring of CREs, which becomes instructive for understanding and modeling *C-MYC*-related developmental processes and associated diseases.

## Methods

### Somatic cell culture

Both *O. aries* (sheep) and *B. taurus* (cattle) ear skin fibroblasts were derived from adult males and cultured in high glucose DMEM (Gibco) supplemented with 10% fetal bovine serum as described by Moura et al*.*^12^. Fibroblasts cultures were passaged by 0.25% trypsin/EDTA (Gibco) when dishes became confluent (passage zero) and subject to 1:3 splits within seven-day intervals. Fibroblasts samples (~ 1.0 × 10^6^) were dissociated by 0.25% trypsin/EDTA, washed twice in 500 µL Phosphate Buffered Saline (PBS) by centrifugation at 500*g* for 5 min. and cell pellets were resuspended in 200 µL PBS without calcium and magnesium (Gibco). Further, cell suspensions were snap-frozen in N_2_ (− 196 °C), and stored at − 80 °C. Total RNA extraction used confluent dishes of early passage fibroblasts (passages two and three).

### Total RNA extraction and cDNA synthesis

Total RNA extraction was carried out using Reliaprep RNA Cell Miniprep (Promega), following the manufacturer instructions. Total RNA was quantified using Nanodrop 2000C (Thermo Scientific) to determine 260/280 and 260/230 ratios and further quantified using Qubit (Thermo Scientific) for cDNA synthesis. The RNA was evaluated by electrophoresis with 1.0% agarose gels in 0.5× TBE buffer under 80 V and 120 A for 40 min.

The reverse transcription (RT) reaction was performed after total RNA extractions using 1.0 µg of total RNA per sample. The procedure was performed with QuantiTect Reverse Transcription Kit (Qiagen). Firstly, residual genomic DNA was removed by the gDNA elimination reaction (7× gDNA wipeout buffer, 1.0 µg total RNA, and ultra-pure H_2_O; 14 µL of total reaction) at 42 °C for 2 min., and transferred to 4 °C. Secondly, the previous reaction mixed to the RT reaction (4 µL 5× Quantscript RT buffer, 1 µL RT primer mix, and 1 µL Quantiscript RT) was kept at 42 °C for 30 min. Finally, samples incubated at 95 °C for three min., and stored at − 20 °C.

### Reverse transcription quantitative PCR (RT-qPCR)

The experiment followed the Minimum Information for Publication of Quantitative Real-Time PCR Experiments (MIQE) guidelines to increase both the transparency and reliability of the RT-qPCR data^50^.

Primers were designed using the strategy outlined by Moura et al*.*^51^. Briefly, reference mRNA sequences were retrieved from GenBank (https://www.ncbi.nlm.nih.gov/genbank/) from selected mammalian species (*Capra hircus*, *O. aries*, and *B. taurus*) and used as templates to design multi-species qPCR primers (see Table 2). Primers were designed using Primer-BLAST (https://www.ncbi.nlm.nih.gov/tools/primer-blast/) and further selected using Primer3plus (https://www.bioinformatics.nl/cgi-bin/primer3plus/primer3plus.cgi). Primer amplification efficiency (E = 10^−1^/slope), correlation coefficient (R^2^), and interception (y) were determined using the standard curve method using cDNA serial dilutions, i.e., 10^0^—non-diluted samples, 10^−1^, 10^−2^, 10^−3^, and 10^−4^^12^.

**Table 2:** Primers used in the study for reverse transcription quantitative PCR (RT-qPCR).

Gene symbol	GenBank access number (species)	Primer sequences [forward (F) and reverse (R)]	Amplicon size (bp)	Refs
**Reference genes**				
*ATP1A1*	NM_001009360.1 (*Ovis aries*)	F: GCA GGG GAT GAA GAA CAA GA R: GAG AAG CGA GTA GGG GAA GG	154	^12^
*RPL19*	gi94966830 (*Bos taurus*)	F: ATG AAA TCG CCA ATG CCA AC R: GGC AGT ACC CTT TCG CTT ACC	167	^51^
*UBB*	NM_001009202.1 (*Ovis aries*)	F: GCA TTG TTG GGT TCC TGT GTC R: CAC GAA GAT TTG CAT TTT GAC	98	^51^
**Target gene**				
*CDK9*	XM_012147609.2 (*Ovis aries musimon*)	F: GCA AAG CAG TAC GAC TCG GT R: GCC TTA AAC ACC TCC CCG AA	104	This study
*CMYC*	NM_001046074.2 (*Bos taurus*)	F: CCA GTA GCG ACT CTG AGG AAG R: TGT GAG GAG GTT TGC TGT GG	135	This study
*RONIN (THAP11)*	NM_001104994 (*Bos taurus*)	F: CAC GGG AGA AGA CGT TAA GC R: GGA GCC AGT ATC AGG GAA GC	187	^51^
*RXRβ*	NM_001083640.1 (*Bos taurus*)	F: TGG GAG CCA TCT TCG ATA GGG T R: CCT TGG CAT CTG GAT TGA ACA GAA	120	This study
*TBX3*	XM_004017387.2 (*Ovis aries*)	F: CTG CTA CTG GGG AAC AGT GG R: GGG AAG GCC AAA GTA AAT CCG	100	This study
*TCF3*	XM_010807251.1 (*Bos taurus*)	F: GTG AGA AGC CCC AGA CCA AA R: GGG TTC AGG TTA CGC TCT CG	94	This study

Refs: references.

The RT-qPCR reactions were carried out using SYBR green system in a Line Gene 9660 FQD-96A real-time PCR (Bioer). The analysis relied on three biological replicates and three technical replicates. The reaction was composed of 1.0 µL cDNA, 2.5 µM primers, 1× Go Taq qPCR Master Mix (Promega), and ultra-pure H_2_O. The reactions were performed under strict conditions (initial denaturation at 95 ºC for 2 min., 40 PCR cycles at 95 ºC for 15 s, 58 ºC for 30 s, and 72 ºC for 30 s). Melting curves were analyzed in 65–95 ºC for 20 min. after the PCR cycles. The RT-qPCR data normalization was carried out using the RGs *ATP1A1*, *RPL19*, and *UBB*^12^. Expression levels of candidate genes evaluated were based upon the number of cycles required for reaching a fixed threshold (quantification cycle—Cq) during the exponential phase of the PCR assay. The relative gene expression levels were evaluated with the REST tool (version 2.0.13) that relies on the 2(−ΔΔCT) method^52^. The supplementary information section contains the raw RT-qPCR data (see Supplementary Table 1) and the results from the REST analysis (see Supplementary Table 2).

### Bioinformatics

The DNA sequences of *C-MYC* gene orthologs were retrieved from GenBank in July 2019. Exons were annotated manually and a 2.5 kb upstream sequence from the transcription start site of each *C-MYC* gene orthologs. Conserved TFBS in *C-MYC* gene orthologs (the complete coding sequences and the additional 2.5 kb DNA upstream sequence) obtained from ConSITE^53^ using the ORCA alignment method and selecting the “all transcription factor profiles” option (https://consite.genereg.net/cgi-bin/consite). Additional TFBS were predicted in *C-MYC* loci using PROMO 3.0^54^. The TFBS search in PROMO was limited to *C-MYC* [T00140], *RXRβ* [T01332], and *TCF3* [T02857] using version 8.3 of TRANSFAC (https://alggen.lsi.upc.es/recerca/menu_recerca.html).

The identification of *C-MYC* mRNA sequences (isoforms) was based on the most recent reference genome assemblies of *B. taurus* (ARS_UCD1.2)^55^ and *O. aries* (oar_rambouillet_v1.0; https://www.hgsc.bcm.edu/other-mammals/sheep-genome-project) using the genome data viewer (https://www.ncbi.nlm.nih.gov/genome/gdv/). The number and identity of *B. taurus* and *O. aries C-MYC* isoforms were retrieved from the Ensembl genome browser (m.ensembl.org). *C-MYC* mRNA sequences obtained from GenBank (RefSeq). The mRNA sequence alignment was carried out using MUSCLE (https://www.ebi.ac.uk/Tools/msa/muscle/)^56^. The mRNA regulatory sequences/motifs were annotated manually. The prediction of RBP binding sites and non-coding RNA binding sites were performed using RegRNA 2.0^57^ with the search option for all available RNA motifs (https://regrna2.mbc.nctu.edu.tw/detection.html). The N^6^-methyladenosine (m^6^A) messenger RNA methylation prediction was carried out using SRAMP^58^. The m^6^A predictive scores (very high, high, moderate, and low) were calculated by SRAMP using the full transcript mode and the generic (default) model for tissue choice (cuilab.cn/sramp/).

*C-MYC* reference protein sequences were retrieved from GenBank and aligned using MUSCLE. Predicted post-translational modifications (PTM) were retrieved from PhosphoSitePlus (phosphositeplus.org). The Venn diagram was prepared using Venny 2.1 using the default option (https://bioinfogp.cnb.csic.es/tools/venny/index.html). Protein secondary structure was predicted with SMART^59^ using the default setting but including the PFAM domain, signal peptide, and internal repeat options (https://smart.embl-heidelberg.de/). Protein domains were obtained from the literature and annotated manually.

### Ethical approval

The research project was approved by the Ethics Commission on Animal Experimentation (CEUA) under the license 031/2016 at the Federal Rural University of Pernambuco (UFRPE). All experiments were performed in accordance with institutional and national guidelines.

## Supplementary information

Supplementary information

## Data Availability

Correspondence and requests for materials addressed to M.T. Moura. All data presented in the manuscript is available upon reasonable request.

## References

[1] Melé M (2015). Human genomics. The human transcriptome across tissues and individuals. Science.

[2] López-Díez R, Rastrojo A, Villate O, Aguado B (2013). Complex tissue-specific patterns and distribution of multiple RAGE splice variants in different mammals. Genome Biol. Evol..

[3] Ulitsky I, Bartel DP (2013). lincRNAs: Genomics, evolution, and mechanisms. Cell.

[4] Stergachis AB (2014). Conservation of trans-acting circuitry during mammalian regulatory evolution. Nature.

[5] Bartel DP (2018). Metazoan microRNAs. Cell.

[6] Stadhouders R, Filion GJ, Graf T (2019). Transcription factors and 3D genome conformation in cell-fate decisions. Nature.

[7] Brawand D (2011). The evolution of gene expression levels in mammalian organs. Nature.

[8] Breschi A, Gingeras TR, Guigó R (2017). Comparative transcriptomics in human and mouse. Nat. Rev. Genet..

[9] Cardoso-Moreira M (2019). Gene expression across mammalian organ development. Nature.

[10] Chen W (2016). Cross-species analysis of gene expression and function in prefrontal cortex, hippocampus and striatum. PLoS ONE.

[11] Rizos D (2004). Species-related differences in blastocyst quality are associated with differences in relative mRNA transcription. Mol. Reprod. Dev..

[12] Moura MT (2019). Inter-genus gene expression analysis in livestock fibroblasts using reference gene validation based upon a multi-species primer set. PLoS ONE.

[13] Maeso I, Tena JJ (2016). Favorable genomic environments for cis-regulatory evolution: A novel theoretical framework. Semin. Cell Dev. Biol..

[14] Sundaram V, Wysocka J (2020). Transposable elements as a potent source of diverse cis-regulatory sequences in mammalian genomes. Philos. Trans. R. Soc. B.

[15] Wagner GP, Lynch VJ (2008). The gene regulatory logic of transcription factor evolution. Trends Ecol. Evol..

[16] Mikkelsen TS (2010). Comparative epigenomic analysis of murine and human adipogenesis. Cell.

[17] Kunarso G (2010). Transposable elements have rewired the core regulatory network of human embryonic stem cells. Nat. Genet..

[18] Kim J (2010). A Myc Network Accounts for similarities between embryonic stem and cancer cell transcription programs. Cell.

[19] Chappell J, Dalton S (2013). Roles for MYC in the establishment and maintenance of pluripotency. Cold Spring Harb. Perspect. Med..

[20] Eilers M, Eisenman RN (2008). Myc's broad reach. Genes Dev..

[21] Psathas JN, Thomas-Tikhonenko A (2014). MYC and the art of microRNA maintenance. Cold Spring Harb. Perspect. Med..

[22] Varlakhanova NV, Knoepfler PS (2009). Acting locally and globally: Myc's ever-expanding roles on chromatin. Cancer Res..

[23] Dominguez-Sola D, Gautier J (2014). MYC and the control of DNA replication. Cold Spring Harb. Perspect. Med..

[24] Bretones G, Delgado MD, León J (2015). Myc and cell cycle control. Biochim. Biophys. Acta.

[25] McMahon SB (2014). MYC and the control of apoptosis. Cold Spring Harb. Perspect. Med..

[26] Rahl PB, Young RA (2014). MYC and transcription elongation. Cold Spring Harb. Perspect. Med..

[27] Nakagawa M (2008). Generation of induced pluripotent stem cells without Myc from mouse and human fibroblasts. Nat. Biotechnol..

[28] Chung HJ, Levens D (2005). c-myc expression: Keep the noise down!. Mol. Cells..

[29] Hurlin PJ (2013). Control of vertebrate development by MYC. Cold Spring Harb. Perspect. Med..

[30] Facchini LM, Chen S, Marhin WW, Lear JN, Penn LZ (1997). The Myc negative autoregulation mechanism requires Myc-Max association and involves the c-myc P2 minimal promoter. Mol. Cell Biol..

[31] Kizaki M (1993). Effects of novel retinoic acid compound, 9-cis-retinoic acid, on proliferation, differentiation, and expression of retinoic acid receptor-alpha and retinoid X receptor-alpha RNA by HL-60 cells. Blood.

[32] Zhu CY (2009). Cell growth suppression by thanatos-associated protein 11 (THAP11) is mediated by transcriptional downregulation of c-Myc. Cell Death Differ..

[33] Shah M, Rennoll SA, Raup-Konsavage WM, Yochum GS (2015). A dynamic exchange of TCF3 and TCF4 transcription factors controls MYC expression in colorectal cancer cells. Cell Cycle.

[34] Lüscher B, Larsson LG (1999). The basic region/helix-loop-helix/leucine zipper domain of Myc proto-oncoproteins: Function and regulation. Oncogene.

[35] Sotelo J (2010). Long-range enhancers on 8q24 regulate c-Myc. Proc. Natl. Acad. Sci. USA.

[36] Xiang JF, Yang L, Chen LL (2015). The long noncoding RNA regulation at the MYC locus. Curr. Opin. Genet. Dev..

[37] Swier LJYM, Dzikiewicz-Krawczyk A, Winkle M, Van Den Berg A, Kluiver J (2019). Intricate crosstalk between MYC and non-coding RNAs regulates hallmarks of cancer. Mol. Oncol..

[38] Schmidt D (2010). Five-vertebrate ChIP-seq reveals the evolutionary dynamics of transcription factor binding. Science.

[39] Trono D (2015). Transposable elements, polydactyl proteins, and the genesis of human-specific transcription networks. Cold Spring Harb. Symp. Quant. Biol..

[40] Trizzino M (2017). Transposable elements are the primary source of novelty in primate gene regulation. Genome Res..

[41] Han J (2010). Tbx3 improves the germ-line competency of induced pluripotent stem cells. Nature.

[42] Fang X (2014). The zinc finger transcription factor ZFX is required for maintaining the tumorigenic potential of glioblastoma stem cells. Stem Cells.

[43] Paulin FE, Chappell SA, Willis AE (1998). A single nucleotide change in the c-myc internal ribosome entry segment leads to enhanced binding of a group of protein factors. Nucleic Acids Res..

[44] Blume SW (2003). Inhibition of tumorigenicity by the 5ʹ-untranslated RNA of the human c-myc P0 transcript. Exp. Cell Res..

[45] Meristoudis C (2015). Systematic analysis of the contribution of c-myc mRNA constituents upon cap and IRES mediated translation. Biol. Chem..

[46] Villar D (2015). Enhancer evolution across 20 mammalian species. Cell.

[47] Lu H (2015). Compensatory induction of MYC expression by sustained CDK9 inhibition via a BRD4-dependent mechanism. Elife.

[48] Boffo S, Damato A, Alfano L, Giordano A (2018). CDK9 inhibitors in acute myeloid leukemia. J. Exp. Clin. Cancer Res..

[49] Lehmann FM (2012). Humanized c-Myc mouse. PLoS ONE.

[50] Taylor S, Wakem M, Dijkman G, Alsarraj M, Nguyen M (2010). A practical approach to RT-qPCR-publishing data that conform to the MIQE guidelines. Methods.

[51] Moura MT (2018). Activity of non-canonical pluripotency-associated transcription factors in goat cumulus-oocyte complexes. Livestock Sci..

[52] Pfaffl MW, Horgan GW, Dempfle L (2002). Relative expression software tool (REST) for groupwise comparison and statistical analysis of relative expression results in real-time PCR. Nucleic Acids Res..

[53] Sandelin A, Wasserman WW, Lenhard B (2004). ConSite: Web-based prediction of regulatory elements using cross-species comparison. Nucleic Acids Res..

[54] Messeguer X (2002). PROMO: Detection of known transcription regulatory elements using species-tailored searches. Bioinformatics.

[55] Shamimuzzaman M (2020). Bovine Genome Database: New annotation tools for a new reference genome. Nucleic Acids Res..

[56] Edgar RC (2004). MUSCLE: Multiple sequence alignment with high accuracy and high throughput. Nucleic Acids Res..

[57] Chang TH (2013). An enhanced computational platform for investigating the roles of regulatory RNA and for identifying functional RNA motifs. BMC Bioinform..

[58] Zhou Y, Zeng P, Li YH, Zhang Z, Cui Q (2016). SRAMP: Prediction of mammalian N6-methyladenosine (m6A) sites based on sequence-derived features. Nucleic Acids Res..

[59] Letunic I, Bork P (2018). 20 years of the SMART protein domain annotation resource. Nucleic Acids Res..

