# Supplementary material for: Evolutionary-driven C-MYC gene expression in mammalian fibroblasts
Source: Sci Rep. 2020 Jul 6;10:11056. doi: 10.1038/s41598-020-67391-x (PMC7338511; doi:10.1038/s41598-020-67391-x)
Supplement: Supplementary file 1 — Supplementary information [file 41598_2020_67391_MOESM1_ESM.pdf]

Evolutionary-driven *C-MYC* gene expression in mammalian fibroblasts

Marcelo T. Moura, Roberta L. O. Silva, Ludymila F. Cantanhêde, José C. Ferreira-Silva, Pábola S. Nascimento, Ana M. Benko-Iseppon, Marcos A. Oliveira

Affiliations: [1]Department of Veterinary Medicine, Federal Rural University of Pernambuco - UFRPE, Brazil; [2]Department of Genetics, Federal University of Pernambuco - UFPE, Brazil; * Author for correspondence: marcelotmoura@gmail.com.

Supplementary Information

Supplementary Fig. 1. Three conserved binding sites for *Homo sapiens MYF* in *Ovis aries* and *Bos taurus C-MYC* gene orthologs determined by ConSITE.

Score	Position	Sequence
9.200	5020	sheep_MYC_2.5kb_Upstream
10.617	5020	Bovine_MYC_2.5kb_Upstream

ID MA0055
Name Myf
Class bHLH
Species Homo sapiens
Sequence

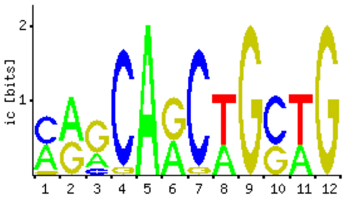

Lit ref
Medline

A	[7	9	4	0	16	7	0	6	0	0	6	0]
C	[8	0	2	15	0	0	15	0	0	10	0	0]
G	[1	7	10	1	0	9	1	0	16	6	0	16]
T	[0	0	0	0	0	0	0	10	0	0	10	0]

Score	Position	Sequence
9.852	5023	sheep_MYC_2.5kb_Upstream
8.656	5023	Bovine_MYC_2.5kb_Upstream

ID MA0055
Name Myf
Class bHLH
Species Homo sapiens
Sequence

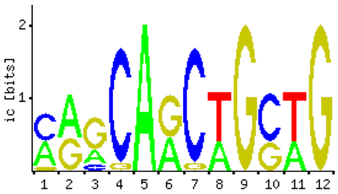

Lit ref
Medline

A	[7	9	4	0	16	7	0	6	0	0	6	0]
C	[8	0	2	15	0	0	15	0	0	10	0	0]
G	[1	7	10	1	0	9	1	0	16	6	0	16]
T	[0	0	0	0	0	0	0	10	0	0	10	0]

Score	Position	Sequence
8.684	5026	sheep_MYC_2.5kb_Upstream
8.907	5026	Bovine_MYC_2.5kb_Upstream

ID MA0055
Name Myf
Class bHLH
Species Homo sapiens
Sequence

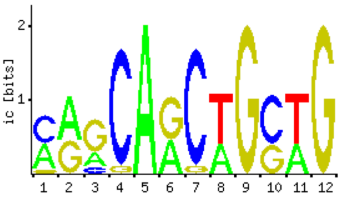

Lit ref
Medline

A	[7	9	4	0	16	7	0	6	0	0	6	0]
C	[8	0	2	15	0	0	15	0	0	10	0	0]
G	[1	7	10	1	0	9	1	0	16	6	0	16]
T	[0	0	0	0	0	0	0	10	0	0	10	0]

Supplementary Fig. 2. The genomic context of *Ovis aries* and *Bos taurus C-MYC* genes. Exons were outlined in gray marking. Transcription factor binding sites are outlined for *C-MYC* (yellow), *RXRβ* (blue), and *TCF3* (red). The *C-MYC* gene sequences (whole gene and 2.5kb upstream the transcription start site) were retrieved from sheep (Oar_rambouillet_v1.0) and bovine (ARS-UCD1.2) reference genomes using the genome data viewer (www.ncbi.nlm.nih.gov/gdv/).

Sheep MYC >NC_040260.1:c27513075-27505598 Ovis aries strain OAR_USU_Benz2616 breed Rambouillet chromosome 9, Oar_rambouillet_v1.0, whole genome shotgun sequence

TTTAAGGAACCGCCCCTTCCTCAGCCGGGTGCTCCCGGACGCCCCTGCCCCCACCCGACCAGCCCGCCCT
CAGCTGGCATCCAGACGCAGCGACCTAGGCTGCCAGTAGAGGGCACACTTACTTTAACTTTCGAAACCTG
GGGCGCGGGTGCAGCTCTGGGAGGGGGCAGGGAAAGACGCTTTGCGGCAAAACTCTGGCTAGGGATTGGT
CACTTGCGTTTGCGGCAAAGACCTGGAGGCTGGAGCAATTTGCAGTCATTAAAACCGACTTGTGCCTTGT
GCCTGATTTGGAGGAAGTTACTACTCTTTTAACAATCGAATGCCGAGCAGCAAACTCAACGGGTAATAAT
TTATCTCAAACACAGGCATGACATAGGCGAGCCCTCCCCGTTCCAAATTTTTTTCACTTTTGGAAGGAGG
GGGAAGGGGCGTGGAAAAGTTATAACGCCCTTGGATGAGGGACTGGGGCTGGGATAGATCGCCTCTGTAT
AATGCAAACTAGTAAATCCCGAGGGGATATGCATTATATATTAAATATAGATTCATTGAGGGAGTGAACA
AATCATGTGTCGTGTTGGGCAAAATTCTAGGCTGACGTGAACATGAAAAGTATACAGATGTACGTGGATT
CTATTATACATACACACTTCACTATGGTATTCAGAAAAAATTTAAGAGTCAGTGAACTAGGAAATCAATG
CCTGAAATTCGGCCAATTTTTAATTGGCTCAAGACACTGCCAGCACACATCCCCCCCCCCCCAAGAAACG
GAAGTAATACTTGGCTCC**TCTCCTTAGAT**GAGAATCGATGCAGTGTGTGTGTGTGTGTGTGTGTGTGT
GTGTGTGTGTGTGATTTCGAGCCCAATGATAAAGAGAAAAGCAGACCCAGAAAGGAAGTCAACGTTCGGT
TTGTCCTAGCAGGAAAGAGTTAACGGTTTTCCTCACCGGGGACTCTGCAGACTCCCCAGGCAGGATCCTC
CGGCTCGCCCCCTTCCCTCTTCTGGGGAGTCTGGTCCTGAGGCTCTCGCCCCCCTCCCCCGGGCTGTATC
CTCCCGTCTAGCGCCTTCTATTTTTCCCCAAACCTGGGAGCCCCAGAGTGGTGCAAACCGGCGCCACGGG
GCGCAAAGAGGATTCGTCTCTCTTCTGAAACCTGGCTGCGGAATTGGGAACTCCGAGAGAGAGGCGCGGG
GGTGGGATGGGGGTTTAGAGGGACGGGGGTGGTCCCCCGCGGCGCGCACCCGCAGGCGCGCTCCTCCCTT
ATTCCAGCTCTGGAACCCGCAGAAAGATCTCAGGGATTAGCGCGTACTGGGTGGGGGGAGGGGCAGGAGG
AACGCTGGCCCCCACGGAGATCCGTGTAGGTCTGCGCGGGCAGAGCCAGGAGTGGGGGTCCTCTGCGACT
TTGGCAGCAATCGGGGGATTCATTGTGGGTGGAAGGTGCGCAGTCGAGAAAGCTGCGCACACCCAGAATG
CATAACACCCCCAACAAATGCAATGGGTGTTTATTCATACCGCGCTCTCCAAGTATACGTGGCAGCGCGT
TGCTGAATTATTTTAGTAATTCCAGGCACCATTTTTCTTCCCC**ATACCTTTGAT**CATGTATCCCATCGAC
ACTTAACCCCCCAGCACACACACACTCAGAGCGCACGCCCATCAATACCCCTTCTTTCCTCCTCTCTTCG
TAGGAGTTATTTTCCAAAGGCTGCCCCTTCCCCGGCCTGTCCACCCGGGACGTGCATGGCGCAGCCGTGG
GTACACGGTGTATTCTCAGCATCTAGGGATGAGCGGTTCTGCCCCCAGATGATTTAAGAACACAGGACAA
GTATGCGGTTTGTCCAACACAGCGCTGCTCCACAGGAACCAAGCAGCAGAGAGACGGTTTGAGCGGGAGC
AAAAGAAAATGGTAGGCGCGCGCAGTTAATTCATGCGGCGCTCTGACCGTGTTTACATCCGAGAGCTCGG
CGCACTGGAGTGCCAGGCTCAAAGAGCTGAATCTCCTCCCCTCCCGCCCTCCCTCCCCACCAGCGCCCCT
CCCGGGTTCCCAAAGCAGAGGGCGGGGGAGACAAAGATCCTCTCTGGTGAGTCCCCGCCCACCAGCCCTT
TATAGGCGAGGGTCTCCGCTGCGGAGGACCCCCGGGCTGCGCATCTCGCGGCTACCGCTGCGGCTGGCTC
GCCGCGGCCGCCGCCTGGCTCCACCACTGCCTGGAAGGGCAGGGCTACTCTGAGGCTTGGCGGGAAAAAG
AACAGAGGAGGGGCCTTACCTTCTCGTATAAAAGCCAGTTTTTTCTGGGCTTTATCTGACTCGCTGTAGT
AATTCCAGCGAGAGGCAGAGGGAGCGAGCGGGCGGGCCCTCCAGGGTGGAAGAGCCGAGCCAGCCTAGCG
AGCGGAGTCGCGCTCCGGGCGCCCGGGGAAGGGAGATCCGGAGTCAAAGGGGGGCTTCTGCGCCTCCAGC
CAGGCCGGCCCCCACCCCACCCTCCCCGCGGACCCCTGCTAGCGGTCCGCCACCCGCGCCGCATCCGCGA
AACTTTGCCCACTGGAACTTACAACACCCGAGCGACACCAAGACTCCCCGGACGCAGAGAAGCTCTTCTG
CCTTTTTGGGGAGACACTTTCCCTCGCCGCAGCCCACGACCCGCGCCTCTGAAAGGCGCTCCTCGCCGCT
TTTTGGACGCTAGATTTCCTTCGGATAGTGGAAATACGGGTGAGCACCCGGACCTATTGGCCTTTTTCAT
TTATTTTTGCTACCGCCTTAACGCCGCGATGAGCAGAACGCCCGAATCGAGTGTCTTTTCTCCCATTCCT
GCGCTATTGACACTTTTCTCAGAGTAGTTGTGGCTGGTCGGGGTGGGGTGGAGAGGAACCAGAACTGGGT
CGGGGTACAGTGACTTGTCAAGATGGGAAGAGAGAGGAAGGCAGAGGGAAACCGGGGGTGCGTTTTCTGA
AAGTAGCCTTTAGAGGTTTTTGCTCGCCTTAGATGGACGCTGACCCCGGCCGGGTGGACATTCATGCTTT
ATTGCATTAATTGCTTTTTTGCCTTTTGTCGGGGAGAAGAAGGGTATGCTTCGCGGTTGACAGAAAACCC
TTTACATCCTGAACTCCTTGGAGTAAGAATTACACATTGCATGTTTGAGCGAAGAGAGCTCCGCAGCCGC
TGACTTTTTCCTGTCTGGAAAAGGGCATTTAAATTTCGGCTTACTGCATTTCTGACAGCCAGAGACAGAC
ACTGCGGCGCGTCCCGCCCGCCGACCCCCTCGGCGTCTCCAACGCTCCCGGCTCCTTTTAGAAGTTGGCC
TTTGACTTTTTTTTAAAGGATAGTAAAATTAAAGATTTTGGTCTACAGAGAGGTTAGGATTCGGTGTTGG
GAAGGCGCGGGGACGGGGAAAAGGAAGCTGGGGCAAAGATGTGTCCGAGTCTCCTGGAATTATTGACTCG
GCGGTGGGGGGGGATGAGGGCAAAATCTCTGCACCTAGCCTTGGCTCCCCGGCCGCCTCCGGGGTCCCCG
CGCGGGTGTCCCTACGCCCCCTGAGATGCGGAGGACGGCCAGGAGCGAGGCACCGGGCTTTTCGAGAACA
GCTGCTACCCTCGGCTGGTGAGGGAGAAGACGCCCGGCTACGGGCGCGGAATCGTAGCAGGGTCTCTGGC

GCAGTTACGTCGCCGTATTGAGTGTTGAAGGGAGACGTCTCTGTTATTATTTAACAC**TCTCCTTTTAT**TT
ATGAGGGTCGGGGGTGGGGGGGTGGGGGGTGGAGAAGGGCTAAAGGCTCGAAGCTGAGCTCGCCACCCCA
GCCGGAGAGAGAAGGAAAGCCCGCAAAGGAAAGAAGGGGGTTGCGCTGGGGGTAGGGATGCGGGAGGCGG
AGAAGGGAGGCTGGGAGGGGCGGCGGCGCCGGCAGGGGAAACAGCGAGACGAGGGCGCGCGAGTGGGAAC
GGGCGCGGCGCTAGGGGCCCGGGAGCGGGAGGGGGCCGAGCGCAACCGCTCCAGCCGCGGCGTCTCTCGC
CGCCTCCTTCAGGTGGCGCAAAACTTTGCGCCCCGGCGTTTGGCAGATCCTATTCCCCACCGCCGCCGCC
GCCTACCCGGGCCTGTGACGGAGGCCAAAGCTGATTTCGATTGCTGGGCGGTCGCGGGGCAGACTCCCGG
GCTTCGCGCTCCAGGCTCCCCGGAGGAGAGCCGAAAAGCCCCGCGGCGCTGCGGAGCTGGTTCGCCAAGT
GTGTGTCTGAGATAGTAGAGGGTCACTCAGAGCGGGCTAAAAGGGTGCCTTTTTTGTCCGTTCCCCACCT
CCCATCAGCCTCATTTGGGGATCGCGCTGGAAGGGGAGTGGTTCTGGATTGTGGCGCGCAGTGCGCGCTG
CGTGGGGGTTTCGGCACCATCCAAGACCCCTCTAACTCAAGACTTGCCCCGTCTTTGTGTGCCCCCTCCG
GCAGGCTACCGCGATGCCCCTCAACGTCAGCTTCGCCAACAGAA**ACTATGACCT**CGACTACGATTCGGTG
CAGCCTTATTTCTACTGCGACGAGGAGGAGAACTTCTACCACCAGCAGCAGCAGAGCGAACTGCAGCCGC
CGGCGCCCAGCGAGGACATCTGGAAGAAATTCGAGCTGCTGCCCACCCCGCCCCTGTCCCCTAGCCGCCG
CTCCGGGCTCTGCTCGCCGTCGTACGTCGCGGTCGCCTCCTTCTCGCCCAGGGGAGACGACGACGGCGGC
GGCGGCAGCTTTTCCTCCGCGGACCGGTTGGAGATGGTGACCGAGCTGCTGGGAGGCGACATGGTGAACC
AGAGCTTCATCTGCGACCCCGACGATGAGACCCTCATCAAAAACATCATCATCCAGGACTGTATGTGGAG
CGGCTTCTCGGCCGCCGCCAAGCTCGTCTCGGAGAAGCTGGCCTCTTACCAGGCTGCGCGCAAAGACGGC
GGCAGCCCGAGCCCCGCCCGCGGGCACGGCGGCTGCTCCACCTCCAGCTTGTACCTGCAGGACCTGAGCG
CCGCCGCCTCTGAATGCATCGACCCCTCGGTGGTCTTCCCCTACCCGCTCAACGACAGCAGCTCGCCCAA
GCCCTGCGCCTCCCCGGACTCCACCGCCTTCTCCCCCTCCTCTGACTCTCTGCTCTCCTCTGCTGAGTCC
TCCCCGCGGGCCAGTCCCGAGCCCCTGGCTCTCCACGAGGAGACCCCACCCACGACCAGCAGCGACTCTG
GTAAGCTGGGACCCTCCAGGCGCGTCAGAGGGGTCGGCTGGCTCTTTCCTATTCTCATTGGCTGCCAACT
CAAGGGGCCATACTTCAGTTGCCCCCCCCCCCCACTGCCCCCCACTGCCTTTTTCTCCTTATATTTGAAA
AAGAAATGTCAGGTCTGGAAGAGTTTGGGAGCTCACCATCCCTGAAACACTGAGCTTTAGTGTTCTTCCC
ACTCCTTCCCCTAAGATCGCCCTAGTGGCCAGTCCTCTCCTTTCCCTTCCCTCCCCTTAGGAATTTCTTT
TGGGTTTTTAAATCTTCTAGCTTATCTAGCAACTCAATCCGCTCCCTCTTACCCCTCTTAAGCATTTTAA
TTGCCCTGGAAGGGGGCGGGGCGAGTCGTCGTTGTGGAGTTAAGAGAGGATTGATCTCCGAGAGTGAATG
AATTACCTCCCTCCTCCCTTCTGAGTTGTTGGGATTTAGTGGACTACAGATCAACAAAAATGAAGAGGGG
CAGTGTACAGAGACCGGGCAGCCTCCCCCTGAGCGCCGGGAGCTAGTGAAAGTGCCTTAAAAGTGTATGG
ACTGAGTTGGGATCTTTGCACCTCTTTTGAACACTAAAAGCCAATCCTTTACAAAATTGGACTTCGGTTT
TTCCTTCCCCCACCTTCTAGGACTTTTGACAAAGCTGCAAGACTTGTTTTTTTTTTTTTTTTTTCTTTTT
GCACTTCCAGTAAGATAGGGAGTTGCTAAAGTTATACCAAAGATTTGCAGCTATCATTTGCAACACCTGA
AGGCTTCTTGGTAAAGTCCCTTGCAAATAGGAGGTGCTTGGGAATGTGCTTTGTCTGGGTGTGTCCAGGG
CCTCATTAAGTCTTAGGTAAGAATTGGCATCAATGCTGTATCCTGGTAAATTGTAATTTTCTTGTCTGTG
CCATAAACCCAGCTGTCATTTTCCTGCCTGAGACTCTCCCTTCTTGAGAAGAAAAGCAGAGATCATTGGC
TACCTGTCTGGGAATAACTTTGTGCCAGGGTCCTTTTTTTTTTTTTTTTTTTAGCCTCACCCAATGGCATG
TGGGATTCTTAGTTCCCCAACTAGGGATCAAAACTTGCCCCCTGTGTTGGAAGCATGGAGTCTTAACCAC
TGGACTGGCAGGGAAGTCCTCTGGGTCCTCTCTTACCTAATAATCCCTTGTATTGGCATAGTATTAATTT
CATCTAGTCATTGATTGCTTTAAGGAAACCGTTGTTAACTGGGTGATTTTTTTCCTTTCTTTCTTAAAGA
GGAAGAACAAGAGGATGAGGAAGAAATTGATGTTGTCTCTGTGGAAAAGAGGCAACCCCCTGCCAAA**AGG**
TCAGAATCGGGGTCACCCTCTGCCGGCAGCCACAGCAAACCTCCTCACAGCCCGTTGGTCCTAAAGAGAT
GC**CACGTG**TCTACGCATCAGCACAATTACGCAGCGCCCCCCTCCACTAGGAAGGACTATCCCGCCGCCAA
GAGGGCTAAGTTGGACAGTGGCAGGGTCCTGAAACAGATCAGCAACAACCGCAAATGTGCCAGCCCGAGG
TCTTCGGACACGGAGGAGAATGACAAGAGGCGGACACACAACGTTTTGGAGCGCCAGAGGAGAAACGAGC
TGAAACGCAGCTTTTTTGCTCTTCGTGACCAGATCCCAGAGTTGGAGAACAATGAAAAAGCCCCCAAAGT
AGTTATCCTTAAAAAAGCCACAGCATACATCCTGTCGGTCCAAGCAGAGGAGCAAAAGCTCATTTCAGAA
AAAGACGTGTTGCGGAAGAGGCGAGAACAGTTGAAACTCAAACTTGAACAGATACGGAACTCTTGCGCCT
AAATTGACCTATTGGAGGGAGGAACTGAACTCCCTCAGGAAATTCTCATTTGTTACTAAGGGAAAGTGAG
GAAAAAGGTTCCTCTGTCAACTCCTTACATAGGAACGTCTTTCATATGCATGAACAACCTCACAACCTTG
GCTGGATCTTTAAGACTGAAAGATTTAGCCATACTATAAACTGCCTCAAATGGAACTTTGGGC**ATAAAAG**
AACTTTTTTTTTTATGCTTACCATTTTTTTTTTTTCCTTTAACAGATTTGTATTTAAGAATTGTTTTTAAAA
ACTCCTGAATTTCACCCAATTTTCCTTTGTAAATATAGCCATTAAATGTAAATAACTTTAATAAAATTTA
TAGTAGGTATACCTACTATATAGTATAATTTTTTTATTTAAGTACATTTTCCTTTTTAAAGTTGATTTTT
TTTCTATTGTTTTTAGAAAAAATAAAATACCTTCAAATATATAATTGAGCCAAATCTT

Bovine MYC >NC_037341.1:c12662116-12653171 Bos taurus isolate L1 Dominette 01449 registration number 42190680 breed Hereford chromosome 14, ARS-UCD1.2, whole genome shotgun sequence

TTGGGTCATCTCGAAAGATATTATTAGTTTTAAATTCCCTTGCCATTTCTCAACCCAGAAAATGTCTCTT
CT**AGGTCAAATG**GCTCTTTAAAATTACTAAAAGAAAGATAGAGAGGTCCCTTAAGACAAGCCTTGCTGAG
ACATGGCTTTCCATTTCTTCGGCTGTGAAATGGGATGGCCCCTACCTTATTTGGAAGCACAAAACTCCAG
GCTGCTACTTGAAAATAACACCACAGTTGATACTACTAGCCTGGGAGAGAAGGCTGAGTCCTGAACCCAC
CAACAGCATCTCTGCTTGGGGTTCACATCACCTACACATGGCTGGCATGGTTTGGTAGTCAGACCTTAAG
ACAGGTATAACAGCCATCTTTAGCAACTTTCAGAAACTACCATCTG**AGGTCAAACT**AGA**CCTACTTTTAT**
TGTTGTTGCTTGATTTATTGGTTTTGAATTTGTGTGTGTGTGTGTGTGTGTTTCTTGCCACAGTAGAGAG
CAGTCATTCAGGCTCCATTCAGTCATTCAGATTCCTAGGAAAACAACCCAAGAGACCTCTTCCAGGAAGG
AGTTCCTGCTGTCACGTTTCCTGAGGATCTATCAGGTGATAAGACACTGTCAAGCATCCTTCTGGTCACA
GAGACCTATCTGAAATATTCACACTCATTAAACATGCTATGTAAATTTCCTGCTCATTTACTTCCACCTT
TATCGCTCA**GTTGTTTTGAT**ATGAAAGCTTTTTATTAAAATGATCCTTTACACTTTTTTCCCAGTGGAAT
CCCAAATGCCCCCCTTCATCGTTATGAAAACATCTGGCTCTTCACCAATTTTTTTTTTCATTTAAATAA
AGCAGTAAGCAAAACTAACTCCTCACCTTTCCCTTTGACTCCACTTCCCCAATAAATCTAACGTCTTTCT
CTAAAACTTGGTGGCTAGATGATTGGTTTCCACGGTGTGTAGTCTAATGCGTGTTTTGCTGCTTTAGAAT
TTTGCTGGAATATTTTTCATGTGTATACTTGTTAATTATTTCACCGTTTCCCATAAATGGAATCAGCTCT
CTATAGGGTGATGTTCGTTGTCTGTAAGTGCCTGCGTGCTCAGTTGCTCAGTCATGTCCGACTCTGCGAC
CCCATGGATGATAACCTGCCAGGCTCCTCTGTCCACGGGATTTCTTCAGGCAAGAATACTGGAATGGGTT
GCCATTGCCGCCTTCAGGGGATCTTCTCGACTCAGGGATCGAACCTGCATCTCCTGCGTCTCCCGCATTG
GAAAGCAGATTCACCACTTAGCCACCTAGGAAGCCTGACCGTGTAGCTATGGGTTAATTTTCACCACCTC
TTATGCAGTTGGACAGCCGCCTCTGAACCACTCTTCTCTCCAGTGATTCCTCTTCTTTCCACCTCTCTGC
AGTTGGCCTGAAGGAATCATAAAACGGCGGCTGGAAACTCAATTTAAGGAACCGCCCCTTCCTCAGCCCG
GTGCTCCGGGACGCCCCTGCCCCCACCCGACCCGCCAGCCCTCAGCTGGCATCCGGACGCAGCGACCTAG
GCTGCCAGTAGAGGGCACACTTACTTTAACTTTCGCAAACCTGGGGCGCGGGTGCAGCTCTGGGAGGGGG
CAGGGAAAGACGCTTTGCAGCAAAACCCTGGCTAGGGATTGGTCACTTGCGTTTTCGGCAAAGACCTGGA
GGCTGGAGGAATTTGCAGTCCTTAAAACCGACTTGTGCCTTGTGCCTGATTTGGAGGAAGTTACTACTCT
TTTAACAATCGAACGCCGAGCAGCAAACTCAACGGGTAATAATTTATCTCAAACACAAGCATGACATAGG
CGAGCCCTCCCTGTCCCGAATTTTTTTTTCACTTTTGGAAGGAGGGGGAAGAGGCGTGGAAAAGTTTACT
TATAACGCCCTTGGATGAGGGACCGGGGCTGGGATAGAGCCTCTGTATAATGCAGAATAGTAAATCCCTA
GGGGATATGCATTATATGTTAAATATAGATTCATTGAGGGAGTGAACAAATCATGTGTCGTGTTGGGCAA
AATTCTAGGTCGACGTGAACATGAAAAGTATACAGATGTACGTGGATTCTATTATACATACACACTTACT
ATGGTATTCAGAAAAAAATTTAAGAGTCAGTGAGCTAGGAAATCAATGCCTGAAATTCGGCCAATTTTTA
ATTGGCTCAAGACACTGCCAGTCCCCCTCCACCCCAAGAAACGGAAGTAATACTTGGCTCC**TCTCCTTAG**
ATGAGAATCGATGCATTGTGTGTGTGTGTGTGTGTGTGTGTGTGTGTGTGTGTGTGATTTCGAGTCCAAT
GATAAAGAGAAAAGCAGACCCAGAAAGGAAGTCAACGTCCGGTTTGTCCAAGCAGGAAAGAGTTAACGGT
TTTCCTTACCGGGGACTCTGCCGACTCCCCAGGCAGGATCCGCCGGCTCGCCCCCTTCCCTCTTCTGGGG
AGTCTGGTCCCGCGGCTCGCGCCCCCCTCCCCCGGGCTGTATCCTCCCGTCTAGCGCCTTTCATTTTTCC
CCAAACCTGGGAGCCCAAGAGTGGTGCAAACCGGCGCCACGGGGCGCAAAGAGGATTCGTCTCTTTTCTG
AAACCTGGCTGCGGAATTGGGAACTCCGAGAGAGAGGCGCGGGGGTGGGATGGGGGTGTAGACGGACGGG
GGTGGTCCCCCGCGGCGCGCACCCGCGGGCGCGCTCCTCCCTTACTCCAGCTCCGGAACCCGCAGAAAGA
TCTCAGGGATTAGCGCGTACCGCGGGGAGGCTGGGGGAGAGGAACGCTGGCCCCCGCGGAGATACGTGTA
GGTCTGCGCGGGCAGGGCCAGGAGTGGGGGCCCTCTGAGACTTTGGCAGCAATCGGGGGATTCATTGTGG
GTGGAAGGTGCCCAGTCGAGAAAGCTGCGCACACCCAGAATGCATAACACCCCCAACAAATGCAATGGGT
GTTTATTCACAACGCGCTCTCCAAGTATACGTGGCAGCGCGTTGCTGAATTATTTTAGTAATTCCAGGCA
CCATTTTTCTTCCGTATACCTCTGATCAGGTATCCCATTGACACTTAAAAAACTCCCCGGCAAACACACA
CTCAGAGCGCGCGCCCATCAATACCCCTTCTTTCCTCCTCTCTTCGCAGGAGTTATTTTCCAAAGGCTGC
CCCTTCCCCGGCCTTAGGGAGGCGCCCTTCCACCCGGACGTGCATGGCGCAGCCGTGGGTACACGGTGTA
TTCTCAGCGTCGAGGGATCAGCGGTTCTGCCCCAGATGATTTAAGAACACAGGACAAGTATGCGGTTTGT
CCAACACAGCGCTGCTCCAGAGGAACCAAGCAGCGCAGAGAGACGGTTTGAGCGGGAGCAAAAGAAAATG
GTAGGCGAGCGCAGTTAATTCATGCGGCGCTCTGACCGTGTTTACACCCGAGAGCTCGACGCAATGGAGT
GCCAGGCTCAAAGAGCTGAATCTCCTCCCCTCCTCACCAGCGCCCCTCCCGGGTTCCTAAAGCAGAGAGC
GGGGGAGACAGAAAAAAGATCCTCTCTGGTGAGTCCCCGCCCACCAGCCCTTTATAGGCGAGGGTCTCCG
CTGCGGAGGACCCCCCGGCTGCGCATCTCGCGGCTACCGCTGCGGCTGGCTCGCCGCGGCCGCCGCCTGG
CTCCACCACTGCCTGGAAGGGCAGGGCTACTCTGAGGCTTGGCGGGAAAAAGAACAGAGGGGAGGGGCCG
TACCTTCTCGGTATAAAAGCCAGTTTTCTGGGCTTTATCTGACTCGCTGTAGTAATTCCAGCGAGAGGCA
GAGGGAGCGTGCGGGCGGGCCCTCCAGGGTGGAAGAGCCGAGCTAGCCTAGCGAGCGGAGTCGCGCTCCG
GGCGCCCGGGGAAGGGAGATCCGGAGTCAAAGGGGGGCTTCTGCGCCTTCAGCCCGGCCGGCCCCCACCC
CACCCACCCCGCGGACCCCTGCTAGCGGTCCGCCACCCGCGCCGCATTCGCGAAACTTTGCCCACTGGAA
CTTACAACATCCGAGCGACACCAAGACTCCCCGGACGCAGAGAAGCTCTTCTGCCTTTTTGGGGAGACAC
TTTCCCCCGCCGCTGCCCACGACCCGCGCCTCTGAAAGGCGCTCCTCGCCGTTTTTTGGACGCTAGATTT
CCTTCGGATAGTGGAAATACGGGTGAGCACCCGGACCTATTGGCCTTTTTCATTTATTTTTGCTACCTCC
TTAACGCCGCGATGAGCAGAACGCCCGAATCGAGTGTCTTTTCTCCCATTCCTGCGCTATTGACACTTTT
CTCAGAGTAGTTGTGGCTGGTCGGGTTGGAGTGGAGAGGAACCAGAACTGGGTCGGGGTACAGTGACTTG

TCAAGATGGGAAGAGAGAGGAAGGCAGAGGGAAACCGGGGGTGCGTTTTCTGAAAGTAGCCTTTAGAGGT
TTTTCCTCGCCTTAGATGGACGCTGACCCCGGCCGGGTGGACATTCATGCTTTATTGCATTAATTGCTTT
TTTGCCTTTTGTCGGGAAGAAGAAGGGTATGCTTCGCGGTTGGCAGAAAACCCTTTACATCCTGAACTCC
TTGGAGTAAGAATTACACATTGCATATGTGAGCGAAGAAAGCTCCGCAGCCGCTGACTTTTCCCTGTCTG
GAAAAGGGCATTTAAATTTCGGCTTACTGCATTTCTGACAGCCGGAGACAGACACTGCGGCGCGTCCCAC
CGCCGATCCCCTCGGCGTCTTCAACGCTCCCGGCTCCTTTTAGAAGTTGGCCTTTGGCTTTTTTTTTTTT
TTTAAGGATAATAAAATTAAAGATTTTGGTCTACAGAGAGGTTAGGATTCGGTGTTGGGAAGGCGCGGGG
ACGGGGAAAAGGAAGGTGGGGCAAAGATGTGTCCGAGTCTCCTGGAATTATTGACTCGGGGTCGGAGGGA
ATGAGGGCAAAATCTCTGCACCCAGCCTTGGCTCCCCGGCCGCCTCCGGGGTCCCCGCGCGGGTGTCCCT
GCGCCCCCTGAGATGCGGAGGACGGCCAGGAGCGGGGCTCCGGGCTTTTCGAGAACAGCTGCTACCCTCG
GCGGGTGAGGGGGAAGACGCCAGGCTCCGGGCGCAGAATCGTAGCAGGGTTTCTGGCGCAGTTGGGTCGC
CGTATTGAGTGTTGAAGGGAGACGTCTCTGTTATTATTTAACAC**TCTCCTTTTAT**TTATGGGGGGGGGGG
GGGGCTGGTTGGAGAAGGGCTAAGGCTCAAAGCTGAGCTAGCCACCCCAGCCGGAGAGAGAAGGAAAGCC
CGCAAAGGAAGGAAGGGGGGCGCGCTGGGGGTAGGGATGCGGGAGGCGGAGAAGGGAGGCTGGGAGGGGC
GGCGGCGCCGGCAGGGGAAGCAGCGAGACGAGGGCGCGCGAGTGGGAACGGGCGCGGCGCTAGGGACCCG
GGAGCGGGAGGGGGCTGAGCGCAACCGCTCCAGCCATGGCGCCTCTCGCCGCCTCCTTCAGGTGGCGCAA
AACTTTGCGCCTCGGCGTTTGGCAGATCCTATTCTCCACCGCCGCCGCTGCCACCGCCGCCGCCGCCTCC
CCCGGCCTGTGACGGAGGCCAAAGCTGATTTCGATTGCTCGGCGGTGGCGGGGCAGACTCCCGGGCTTCG
CGCTCCAGGCTCCCCGCCGGGGAGCAGAAAAGCCCCGCGGCGCTGAGGAGCTGGTTCGCCAAGTGTGTGT
CTGAGATAGTAGAGGGTAACTAAGAGCGGGCTAAAAGGGTGCCTTTTTTGTCCGTTCCCCACCTCCCATC
AGCCCCATTTAGGGATAGCGCTGGAAGGGGAGTGGTTCTGGATTGTGGCGCGCGGCGCGCGGTGCGCGCT
GCGTGGGGTTTCAGCACCATCCAAGACCCCTTTAACTCAAGACTTGCCCCGTCTTTGTGTGCCCCCTCCG
GCAGGCTTGCAGCGATGCCCCTCAACGTCAGCTTCGCCAACAAGA**ACTATGACCT**CGACTACGATTCGGT
GCAGCCTTATTTCTACTGCGACGAGGAGGAGAACTTCTACCACCAGCAGCAGCAGAGCGAACTGCAGCCG
CCGGCGCCCAGCGAGGATATCTGGAAGAAATTCGAGCTGCTGCCCACCCCGCCCCTGTCCCCTAGCCGCC
GCTCCGGGCTCTGCTCGCCGTCGTACGTCGCGGTCGCCTCCTTCTCGCCCAGGGGAGACGACGACGGCGG
CGGCGGCAGCTTCTCCTCAGCGGACCAGTTGGAGATGGTGACCGAGCTACTAGGAGGCGACATGGTGAAC
CAGAGCTTCATCTGCGACCCCGACGATGAGACCCTCATCAAAAACATCATCATCCAGGACTGTATGTGGA
GCGGCTTCTCGGCCGCCGCCAAGCTCGTCTCGGAGAAGCTGGCCTCTTACCAGGCTGCGCGCAAAGACGG
CGGCAGCCCGAGCCCCGCCCGCGGGCACGGCGGCTGCTCCACCTCCAGCTTGTACCTGCAGGACCTGAGC
GCCGCCGCCTCCGAATGCATCGACCCCTCGGTGGTCTTCCCCTACCCGCTCAACGACAGCAGCTCGCCCA
AGCCCTGCGCTTCCCCGGACTCCACCGCCTTTTCTCCGTCCTCTGACTCTCTGCTCTCCTCTGCTGAGTC
CTCCCCGCGGGCCAGTCCCGAGCCCCTGGCGCTCCATGAGGAGACCCCACCCACGACCAGTAGCGACTCT
GGTAAGCTGGGACCCTCCAGGCGCGTCAGAGGGGTCGGCTGGCTGTCTTTCCTATTCTCATTGGCTGCCA
ACTCAAGGGGCCACACTTCAGTTGCCCCCACTCCCCGCCCCCCGCCCCCAGCCTTTCCCCCCTTATATTT
GGAAAAGAAATGGAAAGTCTGGAAGGATCTGGGAGCTCACCATCCCTGAAACACTGAGCCTTAGTGTTCT
TCCCACTCCTTCCCCTAAGATAGCCCTAGTGGCCAGTCCTCTCCTTTCCCTTCCCTCCCCTTAGGAATTT
CTTTTGGGTTTTTAAATCTTCTAGCTTATCTAGCAACTCAATCCGCTCCCTCTTACCCCTCTTAAGCATT
TTAATTGCCCTGGAAGGGGGCGGGGCGAGTCGTCGTCGTGGAGTTAAGAGAGGATTGATCTCTCCGAGAG
TGAATGAATTACCTCCCTCCTCCCTTCAGAGTTGTTGGGATCTAATGGACTACAGATCAACAAAAATGAA
GAGAGTCAGTGTACAGAGACCGGGCAGCCTCCCTCCTGAGCGCCGGGAGCTAGTGAAAAGTGCCTTAAAA
GTGTATGGACTGAGTTGGGATCTTTGCACCTCTTTTGAACACTTAAAAGCCAGACCTTTCCAAAATTGGA
CTTCGTTTTTTCCTTCCCCCACCTTCTAGGACTTTTGGCAAAGCTGCAAGACTTTTTTTTTTTCTTTTTG
CACTTCCAGTAAGATAGGGGGTTGCTAAAGTTATACCAAAGATTTGCAGCTATCATTTGCAACACCTGAA
GGGTTCTTGGTAAAGATAAAGTCCCTTGCAAATAGGAGGTGCTTGGGAATGTGCTTTGTCTGGGTGTGTC
CAGGGCCTCATTAAGTCTTAGGTAAGAATTGGCATCAATGCTGTATCCTGGTAAATTGTAATTTTCTTGT
CTGTGCCATAAACCCAGCTGTCATTTTCCTCCCTGAGACTCTGTCTTCTTGAGAAGAAAAGCAGAGATGA
TTGGCTACCTGTCTGGGAATAACTTTGTGCCAGGGTCCTTTTTATTTTTTTAGCCACACCCAATGGCATG
TGGGATGCTTAGTTCCCCAACCAGGGATCAAAACTTGCCCCCTGTGTTGGAAGCATGGAGTCTTAACCAC
TGGACTGGCAGGGACGTCCTCCGGGTCCTCTCTTACCTAATAATCCTTTGTATTGGCATAGCATTAATTT
CATCTGGTCACTGATTGCTTTAAGGAAACCGTTGTTAACTGGGTGATTTTTTCTTTCTTTCTTAAAGAGG
AAGAACAAGAGGATGAGGAAGAAATTGATGTTGTTTCTGTGGAAAAGAGGCAGCCCCCTGCCAAA**AGGTC**
AGAATCGGGGTCACCCTCTGCCGGCAGCCACAGCAAACCTCCTCACAGCCCGTTAGTCCTAAAGAGATGC
CACGTGTCTACCCATCAGCACAATTACGCAGCGCCCCCCTCCACTAGGAAGGACTATCCCGCCGCCAAGA
GGGCTAAGTTGGACAGTGGCAGGGTCCTGAAACAGATCAGCAACAACCGCAAATGTGCCAGCCCGAGGTC
TTCGGACACGGAGGAGAATGACAAGAGGCGGACACACAACGTTTTGGAGCGCCAGAGGAGAAACGAGCTG
AAACGCAGCTTTTTTGCTCTTCGTGACCAGATCCCAGAGTTGGAGAACAATGAAAAAGCCCCCAAGGTAG
TTATCCTTAAAAAAGCCACAGCGTACATCCTGTCGGTCCAAGCAGAGCAGCAAAAGCTCAAGTCAGAAAT
AGACGTGTTGCAGAAGAGGCGAGAACAGTTGAAACTCAAACTTGAACAGATACGGAACTCTTGCGCCT**AA**
ATTGACCTATTGGAGGGAGGAACTGGACTCCCTCAGGAAATTCTCATTTGTTACTAAGGGAAAGTGAGGA
AAAAGGTTCCCTCTGTCAACTCCTTACATAGGAACATCTTTCATATGCATGAACAACCTCACAACCTTGG
CTGGATCTTTAAGACTGAAAGATTTAGCCATACTATAAACTGCCTCAACTTTGGGC**ATAAAAGAACT**TTT
TTTTTATGCTTACCATTTTTTTTTTTCCTTTAACAGATTTGTATTTAAGAATTGTTTTTAAAAACTCCTGA
ATTTCACCCAATTTTCCTTTGTAAATATAGCCATTAAATGTAAATAACTTTAATAAAATTTATAGTAGGT
ATTCCTACTATAATATAGGTATAATTTTTTTATTTAAGTACATTTTCCTTTTTAAAGTTGATTTTTTTTC
TATTGTTTTTAGAAAAAATAAAATATCTTCAAATATATAATTGAGCCAAATCTTAA

Supplementary Fig. 3. Alignment of *Ovis aries* and *Bos taurus C-MYC* reference mRNA orthologs using MUSCLE. *O. aries* mRNA (NM_001009426.1), *B. taurus* mRNA (NM_001046074.2), *B. taurus* X1 (XM_005215324.4), and *B. taurus* X2 (XM_010811929.3).

```
XM_005215324.4 CTAGCGCCTTTCATTTTTCCCCAAACCTGGGAGCCCAAGAGTGGTGCAAACCGGCGCCAC
XM_010811929.3 ------------------------------------------------------------
NM_001009426.1 ------------------------------------------------------------
NM_001046074.2 -----GACTCGCTGTAGTAATTCCAGCGAGAGGCAGAGGGAGCGTGCGGGCGGGCCCTCC

XM_005215324.4 GGGGCGCAAAG--AGGATTCGTCTCTTTTCTGAAACCTGGCTGCGGAATTGGGAACTCCG
XM_010811929.3 ------------------------------------------------------------
NM_001009426.1 ------------------------------------------------------------
NM_001046074.2 AGGGTGGAAGAGCCGAGCTAGCCTAGCGAGCGGAGTCGCGCTCC------GGGCGCCCGG

XM_005215324.4 AGAGAGAGGCGCGGGGGTGGGATGGGGGTGTAGACGGAC---------------------
XM_010811929.3 ------------------------------------------------------------
NM_001009426.1 ------------------------------------------------------------
NM_001046074.2 GGAAGGGAGATCCGGAGTCAAAGGGGGGCTTCTGCGCCTTCAGCCCGGCCGGCCCCCACC

XM_005215324.4 -------------------------GGGGGTGGTCCCCCGCGGCGCGCACCCGCGGGCGC
XM_010811929.3 ----------------------------------------CGCCGC--------------
NM_001009426.1 ----------------------------------CACCCGCGCTGC--ATCCGCGAA---
NM_001046074.2 CCACCCACCCCGCGGACCCCTGCTAGCGGTCCGCCACCCGCGCCGC--ATTCGCGAA---
                                                            **  **
XM_005215324.4 GCTCCTCCCTTACTCCAGCTCCGGAACCCGCAGAAAGATCTCAGGGATTAGCGCGTACCG
XM_010811929.3 -CTCCCCC----------------GGCCTGTGACG-----------------------GA
NM_001009426.1 ACTTTGCC----------CACTGGAACTTACAACA----------------CCCGAGCGA
NM_001046074.2 ACTTTGCC----------CACTGGAACTTACAACA----------------TCCGAGCGA
                **   **                   *
XM_005215324.4 CGGGGAGGCTGGGGGAGAGGAACGCTGGCCCC---CGCGGAGATACGTGAGTTATTTTCC
XM_010811929.3 GGCCAAAGCT----------------GATTTCGATTGCTCGGCGG---------------
NM_001009426.1 CACCAAGACT------------------CCCCGGACGCAGAGAAG---------------
NM_001046074.2 CACCAAGACT------------------CCCCGGACGCAGAGAAG---------------
                    *  **                     *    **   *
XM_005215324.4 AAAGGCTGCCCCTTCCCCGGCCTTAGGGAGGCGCCCTTCCACCC--GGACGTGCATGGCG
XM_010811929.3 -----------------------TGGCGGGGCAGACTCCCGGGCTTCGCGCTCCAGGCT-
NM_001009426.1 ---------CTCTTCTGCCTTTTTGGGGAGACACTTTCCCTCGC--CGCAGCCCACGAC-
NM_001046074.2 ---------CTCTTCTGCCTTTTTGGGGAGACACTTTCCCCCGC--CGCTGCCCACGAC-
                                        * * * * *    * **   *   *     ** *
XM_005215324.4 CAGCCGTGGGTACACGGTGTATTCTCAGCGTCGAGGGATCAGCGGTTCTGCCCCAGATGA
XM_010811929.3 ------------------------------------------------------------
NM_001009426.1 ------------------------------------------------------------
NM_001046074.2 ------------------------------------------------------------

XM_005215324.4 TTTAAGAACACAGGACAAGTATGCGGTTTGTCCAACACAGCGCTGCTCCAGAGGAACCAA
XM_010811929.3 -------------------------------------CCCCGCCG---------------
NM_001009426.1 -------------------------------------CCGCGCCTCT-------------
NM_001046074.2 -------------------------------------CCGCGCCTCT-------------
                                                         *  ***

XM_005215324.4 GCAGCGCAGAGAGACGGTTTGAGCGGGAGCAAAAGAAAATGGTAGGCGAGCGCAGTTAAT
XM_010811929.3 ------------------------GGGAGCAGAA--------------------------
NM_001009426.1 ------------------------GAAAG-------------------------------
NM_001046074.2 ------------------------GAAAG-------------------------------
                                            *  **

XM_005215324.4 TCATGCGGCGCTCTGACCGTGTTTACACCCGAGAGCTCGACGCAATGGAGTGCCAGGCTC
XM_010811929.3 -------AAGCCC----------------------CGCGGCGC---TGAG----------
NM_001009426.1 -------GCGCTC----------------------CTCGCCGCTTTTTGG----------
NM_001046074.2 -------GCGCTC----------------------CTCGCCGTTTTTTGG----------
                             ** *                      * ** **       *
XM_005215324.4 AAAGAGCTGAATCTCCTCCCCTCCTCACCAGCGCCCCTCCCGGGTTCCTAAAGCAGAGAG
XM_010811929.3 ---GAGCTGGTTCGC---------------------------------------------
NM_001009426.1 ---ACGCTAGATTTCCTTC-----------------------------------------
NM_001046074.2 ---ACGCTAGATTTCCTTC-----------------------------------------
                         ***   *  *
XM_005215324.4 CGGGGGAGACAGAAAAAAGATCCTCTCTGGCTTGCAGCGATGCCCCTCAACGTCAGCTTC
XM_010811929.3 --------------------------CAAGCTTGCAGCGATGCCCCTCAACGTCAGCTTC
NM_001009426.1 ------GGATAGTGGAAATA------CGGGC-TACCGCGATGCCCCTCAACGTCAGCTTC
```

```
NM_001046074.2 ------GGATAGTGGAAATA------CGGGCTTGCAGCGATGCCCCTCAACGTCAGCTTC
                                         *  ** * * ************************

XM_005215324.4 GCCAACAAGAACTATGACCTCGACTACGATTCGGTGCAGCCTTATTTCTACTGCGACGAG
XM_010811929.3 GCCAACAAGAACTATGACCTCGACTACGATTCGGTGCAGCCTTATTTCTACTGCGACGAG
NM_001009426.1 GCCAACAGAAACTATGACCTCGACTACGATTCGGTGCAGCCTTATTTCTACTGCGACGAG
NM_001046074.2 GCCAACAAGAACTATGACCTCGACTACGATTCGGTGCAGCCTTATTTCTACTGCGACGAG
               *******  ***************************************************

XM_005215324.4 GAGGAGAACTTCTACCACCAGCAGCAGCAGAGCGAACTGCAGCCGCCGGCGCCCAGCGAG
XM_010811929.3 GAGGAGAACTTCTACCACCAGCAGCAGCAGAGCGAACTGCAGCCGCCGGCGCCCAGCGAG
NM_001009426.1 GAGGAGAACTTCTACCACCAGCAGCAGCAGAGCGAACTGCAGCCGCCGGCGCCCAGCGAG
NM_001046074.2 GAGGAGAACTTCTACCACCAGCAGCAGCAGAGCGAACTGCAGCCGCCGGCGCCCAGCGAG
               ************************************************************

XM_005215324.4 GATATCTGGAAGAAATTCGAGCTGCTGCCCACCCCGCCCCTGTCCCCTAGCCGCCGCTCC
XM_010811929.3 GATATCTGGAAGAAATTCGAGCTGCTGCCCACCCCGCCCCTGTCCCCTAGCCGCCGCTCC
NM_001009426.1 GACATCTGGAAGAAATTCGAGCTGCTGCCCACCCCGCCCCTGTCCCCTAGCCGCCGCTCC
NM_001046074.2 GATATCTGGAAGAAATTCGAGCTGCTGCCCACCCCGCCCCTGTCCCCTAGCCGCCGCTCC
               ** *********************************************************

XM_005215324.4 GGGCTCTGCTCGCCGTCGTACGTCGCGGTCGCCTCCTTCTCGCCCAGGGGAGACGACGAC
XM_010811929.3 GGGCTCTGCTCGCCGTCGTACGTCGCGGTCGCCTCCTTCTCGCCCAGGGGAGACGACGAC
NM_001009426.1 GGGCTCTGCTCGCCGTCGTACGTCGCGGTCGCCTCCTTCTCGCCCAGGGGAGACGACGAC
NM_001046074.2 GGGCTCTGCTCGCCGTCGTACGTCGCGGTCGCCTCCTTCTCGCCCAGGGGAGACGACGAC
               ************************************************************

XM_005215324.4 GGCGGCGGCGGCAGCTTCTCCTCAGCGGACCAGTTGGAGATGGTGACCGAGCTACTAGGA
XM_010811929.3 GGCGGCGGCGGCAGCTTCTCCTCAGCGGACCAGTTGGAGATGGTGACCGAGCTACTAGGA
NM_001009426.1 GGCGGCGGCGGCAGCTTTTCCTCCGCGGACCGGTTGGAGATGGTGACCGAGCTGCTGGGA
NM_001046074.2 GGCGGCGGCGGCAGCTTCTCCTCAGCGGACCAGTTGGAGATGGTGACCGAGCTACTAGGA
               ***************** ***** ******* ********************* ** ***

XM_005215324.4 GGCGACATGGTGAACCAGAGCTTCATCTGCGACCCCGACGATGAGACCCTCATCAAAAAC
XM_010811929.3 GGCGACATGGTGAACCAGAGCTTCATCTGCGACCCCGACGATGAGACCCTCATCAAAAAC
NM_001009426.1 GGCGACATGGTGAACCAGAGCTTCATCTGCGACCCCGACGATGAGACCCTCATCAAAAAC
NM_001046074.2 GGCGACATGGTGAACCAGAGCTTCATCTGCGACCCCGACGATGAGACCCTCATCAAAAAC
               ************************************************************

XM_005215324.4 ATCATCATCCAGGACTGTATGTGGAGCGGCTTCTCGGCCGCCGCCAAGCTCGTCTCGGAG
XM_010811929.3 ATCATCATCCAGGACTGTATGTGGAGCGGCTTCTCGGCCGCCGCCAAGCTCGTCTCGGAG
NM_001009426.1 ATCATCATCCAGGACTGTATGTGGAGCGGCTTCTCGGCCGCCGCCAAGCTCGTCTCGGAG
NM_001046074.2 ATCATCATCCAGGACTGTATGTGGAGCGGCTTCTCGGCCGCCGCCAAGCTCGTCTCGGAG
               ************************************************************

XM_005215324.4 AAGCTGGCCTCTTACCAGGCTGCGCGCAAAGACGGCGGCAGCCCGAGCCCCGCCCGCGGG
XM_010811929.3 AAGCTGGCCTCTTACCAGGCTGCGCGCAAAGACGGCGGCAGCCCGAGCCCCGCCCGCGGG
NM_001009426.1 AAGCTGGCCTCTTACCAGGCTGCGCGCAAAGACGGCGGCAGCCCGAGCCCCGCCCGCGGG
NM_001046074.2 AAGCTGGCCTCTTACCAGGCTGCGCGCAAAGACGGCGGCAGCCCGAGCCCCGCCCGCGGG
               ************************************************************

XM_005215324.4 CACGGCGGCTGCTCCACCTCCAGCTTGTACCTGCAGGACCTGAGCGCCGCCGCCTCCGAA
XM_010811929.3 CACGGCGGCTGCTCCACCTCCAGCTTGTACCTGCAGGACCTGAGCGCCGCCGCCTCCGAA
NM_001009426.1 CACGGCGGCTGCTCCACCTCCAGCTTGTACCTGCAGGACCTGAGCGCCGCCGCCTCTGAA
NM_001046074.2 CACGGCGGCTGCTCCACCTCCAGCTTGTACCTGCAGGACCTGAGCGCCGCCGCCTCCGAA
               ******************************************************** ***

XM_005215324.4 TGCATCGACCCCTCGGTGGTCTTCCCCTACCCGCTCAACGACAGCAGCTCGCCCAAGCCC
XM_010811929.3 TGCATCGACCCCTCGGTGGTCTTCCCCTACCCGCTCAACGACAGCAGCTCGCCCAAGCCC
NM_001009426.1 TGCATCGACCCCTCGGTGGTCTTCCCCTACCCGCTCAACGACAGCAGCTCGCCCAAGCCC
NM_001046074.2 TGCATCGACCCCTCGGTGGTCTTCCCCTACCCGCTCAACGACAGCAGCTCGCCCAAGCCC
               ************************************************************

XM_005215324.4 TGCGCTTCCCCGGACTCCACCGCCTTTTCTCCGTCCTCTGACTCTCTGCTCTCCTCTGCT
XM_010811929.3 TGCGCTTCCCCGGACTCCACCGCCTTTTCTCCGTCCTCTGACTCTCTGCTCTCCTCTGCT
NM_001009426.1 TGCGCCTCCCCGGACTCCACCGCCTTCTCCCCCTCCTCTGACTCTCTGCTCTCCTCTGCT
NM_001046074.2 TGCGCTTCCCCGGACTCCACCGCCTTTTCTCCGTCCTCTGACTCTCTGCTCTCCTCTGCT
               ***** ******************** ** ** ***************************

XM_005215324.4 GAGTCCTCCCCGCGGGCCAGTCCCGAGCCCCTGGCGCTCCATGAGGAGACCCCACCCACG
XM_010811929.3 GAGTCCTCCCCGCGGGCCAGTCCCGAGCCCCTGGCGCTCCATGAGGAGACCCCACCCACG
NM_001009426.1 GAGTCCTCCCCGCGGGCCAGTCCCGAGCCCCTGGCTCTCCACGAGGAGACCCCACCCACG
NM_001046074.2 GAGTCCTCCCCGCGGGCCAGTCCCGAGCCCCTGGCGCTCCATGAGGAGACCCCACCCACG
               *********************************** ***** ******************

XM_005215324.4 ACCAGTAGCGACTCTGAGGAAGAACAAGAGGATGAGGAAGAAATTGATGTTGTTTCTGTG
XM_010811929.3 ACCAGTAGCGACTCTGAGGAAGAACAAGAGGATGAGGAAGAAATTGATGTTGTTTCTGTG
NM_001009426.1 ACCAGCAGCGACTCTGAGGAAGAACAAGAGGATGAGGAAGAAATTGATGTTGTCTCTGTG
NM_001046074.2 ACCAGTAGCGACTCTGAGGAAGAACAAGAGGATGAGGAAGAAATTGATGTTGTTTCTGTG
               ***** *********************************************** ******
```

```
XM_005215324.4 GAAAAGAGGCAGCCCCCTGCCAAAAGGTCAGAATCGGGGTCACCCTCTGCCGGCAGCCAC
XM_010811929.3 GAAAAGAGGCAGCCCCCTGCCAAAAGGTCAGAATCGGGGTCACCCTCTGCCGGCAGCCAC
NM_001009426.1 GAAAAGAGGCAACCCCCTGCCAAAAGGTCAGAATCGGGGTCACCCTCTGCCGGCAGCCAC
NM_001046074.2 GAAAAGAGGCAGCCCCCTGCCAAAAGGTCAGAATCGGGGTCACCCTCTGCCGGCAGCCAC
               *********** ************************************************

XM_005215324.4 AGCAAACCTCCTCACAGCCCGTTAGTCCTAAAGAGATGCCACGTGTCTACCCATCAGCAC
XM_010811929.3 AGCAAACCTCCTCACAGCCCGTTAGTCCTAAAGAGATGCCACGTGTCTACCCATCAGCAC
NM_001009426.1 AGCAAACCTCCTCACAGCCCGTTGGTCCTAAAGAGATGCCACGTGTCTACGCATCAGCAC
NM_001046074.2 AGCAAACCTCCTCACAGCCCGTTAGTCCTAAAGAGATGCCACGTGTCTACCCATCAGCAC
               *********************** ************************** *********

XM_005215324.4 AATTACGCAGCGCCCCCCTCCACTAGGAAGGACTATCCCGCCGCCAAGAGGGCTAAGTTG
XM_010811929.3 AATTACGCAGCGCCCCCCTCCACTAGGAAGGACTATCCCGCCGCCAAGAGGGCTAAGTTG
NM_001009426.1 AATTACGCAGCGCCCCCCTCCACTAGGAAGGACTATCCCGCCGCCAAGAGGGCTAAGTTG
NM_001046074.2 AATTACGCAGCGCCCCCCTCCACTAGGAAGGACTATCCCGCCGCCAAGAGGGCTAAGTTG
               ************************************************************

XM_005215324.4 GACAGTGGCAGGGTCCTGAAACAGATCAGCAACAACCGCAAATGTGCCAGCCCGAGGTCT
XM_010811929.3 GACAGTGGCAGGGTCCTGAAACAGATCAGCAACAACCGCAAATGTGCCAGCCCGAGGTCT
NM_001009426.1 GACAGTGGCAGGGTCCTGAAACAGATCAGCAACAACCGCAAATGTGCCAGCCCGAGGTCT
NM_001046074.2 GACAGTGGCAGGGTCCTGAAACAGATCAGCAACAACCGCAAATGTGCCAGCCCGAGGTCT
               ************************************************************

XM_005215324.4 TCGGACACGGAGGAGAATGACAAGAGGCGGACACACAACGTTTTGGAGCGCCAGAGGAGA
XM_010811929.3 TCGGACACGGAGGAGAATGACAAGAGGCGGACACACAACGTTTTGGAGCGCCAGAGGAGA
NM_001009426.1 TCGGACACGGAGGAGAATGACAAGAGGCGGACACACAACGTTTTGGAGCGCCAGAGGAGA
NM_001046074.2 TCGGACACGGAGGAGAATGACAAGAGGCGGACACACAACGTTTTGGAGCGCCAGAGGAGA
               ************************************************************

XM_005215324.4 AACGAGCTGAAACGCAGCTTTTTTGCTCTTCGTGACCAGATCCCAGAGTTGGAGAACAAT
XM_010811929.3 AACGAGCTGAAACGCAGCTTTTTTGCTCTTCGTGACCAGATCCCAGAGTTGGAGAACAAT
NM_001009426.1 AACGAGCTGAAACGCAGCTTTTTTGCTCTTCGTGACCAGATCCCAGAGTTGGAGAACAAT
NM_001046074.2 AACGAGCTGAAACGCAGCTTTTTTGCTCTTCGTGACCAGATCCCAGAGTTGGAGAACAAT
               ************************************************************

XM_005215324.4 GAAAAAGCCCCCAAGGTAGTTATCCTTAAAAAAGCCACAGCGTACATCCTGTCGGTCCAA
XM_010811929.3 GAAAAAGCCCCCAAGGTAGTTATCCTTAAAAAAGCCACAGCGTACATCCTGTCGGTCCAA
NM_001009426.1 GAAAAAGCCCCCAAAGTAGTTATCCTTAAAAAAGCCACAGCATACATCCTGTCGGTCCAA
NM_001046074.2 GAAAAAGCCCCCAAGGTAGTTATCCTTAAAAAAGCCACAGCGTACATCCTGTCGGTCCAA
               ************** ************************** ******************

XM_005215324.4 GCAGAGCAGCAAAAGCTCAAGTCAGAAATAGACGTGTTGCAGAAGAGGCGAGAACAGTTG
XM_010811929.3 GCAGAGCAGCAAAAGCTCAAGTCAGAAATAGACGTGTTGCAGAAGAGGCGAGAACAGTTG
NM_001009426.1 GCAGAGGAGCAAAAGCTCATTTCAGAAAAAGACGTGTTGCGGAAGAGGCGAGAACAGTTG
NM_001046074.2 GCAGAGCAGCAAAAGCTCAAGTCAGAAATAGACGTGTTGCAGAAGAGGCGAGAACAGTTG
               ****** ************  ******* *********** *******************

XM_005215324.4 AAACTCAAACTTGAACAGATACGGAACTCTTGCGCCTAAATTGACCTATTGGAGGGAGGA
XM_010811929.3 AAACTCAAACTTGAACAGATACGGAACTCTTGCGCCTAAATTGACCTATTGGAGGGAGGA
NM_001009426.1 AAACTCAAACTTGAACAGATACGGAACTCTTGCGCCTAAATTGACCTATTGGAGGGAGGA
NM_001046074.2 AAACTCAAACTTGAACAGATACGGAACTCTTGCGCCTAAATTGACCTATTGGAGGGAGGA
               ************************************************************

XM_005215324.4 ACTGGACTCCCTCAGGAAATTCTCATTTGTTACTAAGGGAAAGTGAGGAAAAAGGTTCCC
XM_010811929.3 ACTGGACTCCCTCAGGAAATTCTCATTTGTTACTAAGGGAAAGTGAGGAAAAAGGTTCCC
NM_001009426.1 ACTGAACTCCCTCAGGAAATTCTCATTTGTTACTAAGGGAAAGTGAGGAAAAAGGTT-CC
NM_001046074.2 ACTGGACTCCCTCAGGAAATTCTCATTTGTTACTAAGGGAAAGTGAGGAAAAAGGTTCCC
               **** **************************************************** **

XM_005215324.4 TCTGTCAACTCCTTACATAGGAACATCTTTCATATGCATGAACAACCTCACAACCTTGGC
XM_010811929.3 TCTGTCAACTCCTTACATAGGAACATCTTTCATATGCATGAACAACCTCACAACCTTGGC
NM_001009426.1 TCTGTCAACTCCTTACATAGGAACGTCTTTCATATGCATGAACAACCTCACAACCTTGGC
NM_001046074.2 TCTGTCAACTCCTTACATAGGAACATCTTTCATATGCATGAACAACCTCACAACCTTGGC
               *********************** ************************************

XM_005215324.4 TGGATCTTTAAGACTGAAAGATTTAGCCATACTATAAACTGCCTC------AACTTTGGG
XM_010811929.3 TGGATCTTTAAGACTGAAAGATTTAGCCATACTATAAACTGCCTC------AACTTTGGG
NM_001009426.1 TGGATCTTTAAGACTGAAAGATTTAGCCATACTATAAACTGCCTCAAATGGAACTTTGGG
NM_001046074.2 TGGATCTTTAAGACTGAAAGATTTAGCCATACTATAAACTGCCTC------AACTTTGGG
               *********************************************      *********
```

```
XM_005215324.4 CATAAAAGAACTTTTTTTTTTATGCTTACCATTTTTTTTTCCTTTAACAGATTTGTATTT
XM_010811929.3 CATAAAAGAACTTTTTTTTTTATGCTTACCATTTTTTTTTCCTTTAACAGATTTGTATTT
NM_001009426.1 CATAAAAGAACTTTTTTTTTTATGCTTACCATTTTTTTTTCCTTTAACAGATTTGTATTT
NM_001046074.2 CATAAAAGAACTTTTTTTTTTATGCTTACCATTTTTTTTTCCTTTAACAGATTTGTATTT
               ************************************************************

XM_005215324.4 AAGAATTGTTTTTAAAAACTCCTGAATTTCACCCAATTTTCCTTTGTAAATATAGCCATT
XM_010811929.3 AAGAATTGTTTTTAAAAACTCCTGAATTTCACCCAATTTTCCTTTGTAAATATAGCCATT
NM_001009426.1 AAGAATTGTTTTTAAAAACTCCTGAATTTCACCCAATTTTCCTTTGTAAATATAGCCATT
NM_001046074.2 AAGAATTGTTTTTAAAAACTCCTGAATTTCACCCAATTTTCCTTTGTAAATATAGCCATT
               ************************************************************

XM_005215324.4 AAATGTAAATAACTTTAATAAAATTTATAGTAGGTATTCCTACTATAATATAGGTATAAT
XM_010811929.3 AAATGTAAATAACTTTAATAAAATTTATAGTAGGTATTCCTACTATAATATAGGTATAAT
NM_001009426.1 AAATGTAAATAACTTTAATAAAATTTATAGTAGGTATACCTACTATA----TAGTATAAT
NM_001046074.2 AAATGTAAATAACTTTAATAAAATTTATAGTAGGTATTCCTACTATAATATAGGTATAAT
               ************************************* *********      *******

XM_005215324.4 TTTTTTATTTAAGTACATTTTCCTTTTTAAAGTTGATTTTTTTTCTATTGTTTTTAGAAA
XM_010811929.3 TTTTTTATTTAAGTACATTTTCCTTTTTAAAGTTGATTTTTTTTCTATTGTTTTTAGAAA
NM_001009426.1 TTTTTTATTTAAGTACATTTTCCTTTTTAAAGTTGATTTTTTTTCTATTGTTTTTAGAAA
NM_001046074.2 TTTTTTATTTAAGTACATTTTCCTTTTTAAAGTTGATTTTTTTTCTATTGTTTTTAGAAA
               ************************************************************

XM_005215324.4 AAATAAAATATCTTCAAATATATAATTGAGCCAAA---------
XM_010811929.3 AAATAAAATATCTTCAAATATATAATTGAGCCAAA---------
NM_001009426.1 AAATAAAATACCTTCAAATATATAATTGAGCCAAATCTT-----
NM_001046074.2 AAATAAAATATCTTCAAATATATAATTGAGCCAAATCTTAAAAA
               ********** ************************
CLUSTAL multiple sequence alignment by MUSCLE (3.8)
```

Supplementary Fig. 4. Reference mRNA sequences of *Ovis aries* and *Bos taurus C-MYC* mRNA orthologs and annotation of regulatory sequences/motifs. Coding sequence (bold letters). Polyadenylation signal (dark blue). Stem loop/AU-rich sequence (purple). 3' UTR octamer (underlined blue). AU-rich sequence element (UUUN[A/U]U] (ASE; underlined red). Internal Ribosome Entry Site (IRES; green). AU-rich element (ARE; orange). AUUUA motifs were also highlighted.

>NM_001009426.1 *Ovis aries MYC* proto-oncogene, bHLH Transcription factor (*MYC*), mRNA

CACCCGCGCUGCAUCCGCGAAACUUUGCCCACUGGAACUUACAACACCCGAGCGACACCAAGACUCCCCG
GACGCAGAGAAGCUCUUCUGCCUUUUUGGGGAGACACUUUCCCUCGCCGCAGCCCACGACCCGCGCCUCU
GAAAGGCGCUCCUCGCCGCUUUUUGGACGCUAGAUUUCCUUCGGAUAGUGGAAAUACGGGCUACCGCG**AU**
GCCCCUCAACGUCAGCUUCGCCAACAGAAACUAUGACCUCGACUACGAUUCGGUGCAGCCUUAUUUCUAC
UGCGACGAGGAGGAGAACUUCUACCACCAGCAGCAGCAGAGCGAACUGCAGCCGCCGGCGCCCAGCGAGG
ACAUCUGGAAGAAAUUCGAGCUGCUGCCCACCCCGCCCCUGUCCCCUAGCCGCCGCUCCGGGCUCUGCUC
GCCGUCGUACGUCGCGGUCGCCUCCUUCUCGCCCAGGGGAGACGACGACGGCGGCGGCGGCAGCUUUUCC
UCCGCGGACCGGUUGGAGAUGGUGACCGAGCUGCUGGGAGGCGACAUGGUGAACCAGAGCUUCAUCUGCG
ACCCCGACGAUGAGACCCUCAUCAAAAACAUCAUCAUCCAGGACUGUAUGUGGAGCGGCUUCUCGGCCGC
CGCCAAGCUCGUCUCGGAGAAGCUGGCCUCUUACCAGGCUGCGCGCAAAGACGGCGGCAGCCCGAGCCCC
GCCCGCGGGCACGGCGGCUGCUCCACCUCCAGCUUGUACCUGCAGGACCUGAGCGCCGCCGCCUCUGAAU
GCAUCGACCCCUCGGUGGUCUUCCCCUACCCGCUCAACGACAGCAGCUCGCCCAAGCCCUGCGCCUCCCC
GGACUCCACCGCCUUCUCCCCCUCCUCUGACUCUCUGCUCUCCUCUGCUGAGUCCUCCCCGCGGGCCAGU
CCCGAGCCCCUGGCUCUCCACGAGGAGACCCCACCCACGACCAGCAGCGACUCUGAGGAAGAACAAGAGG
AUGAGGAAGAAAUUGAUGUUGUCUCUGUGGAAAAGAGGCAACCCCCUGCCAAAAGGUCAGAAUCGGGGUC
ACCCUCUGCCGGCAGCCACAGCAAACCUCCUCACAGCCCGUUGGUCCUAAAGAGAUGCCACGUGUCUACG
CAUCAGCACAAUUACGCAGCGCCCCCCUCCACUAGGAAGGACUAUCCCGCCGCCAAGAGGGCUAAGUUGG
ACAGUGGCAGGGUCCUGAAACAGAUCAGCAACAACCGCAAAUGUGCCAGCCCGAGGUCUUCGGACACGGA
GGAGAAUGACAAGAGGCGGACACACAACGUUUUGGAGCGCCAGAGGAGAAACGAGCUGAAACGCAGCUUU
UUUGCUCUUCGUGACCAGAUCCCAGAGUUGGAGAACAAUGAAAAAGCCCCCAAAGUAGUUAUCCUUAAAA
AAGCCACAGCAUACAUCCUGUCGGUCCAAGCAGAGGAGCAAAAGCUCAUUUCAGAAAAAGACGUGUUGCG
GAAGAGGCGAGAACAGUUGAAACUCAAACUUGAACAGAUACGGAACUCUUGCGCCUAAAUUGACCUAUUG
GAGGGAGGAACUGAACUCCCUCAGGAAAUUCUCAUUUGUUACUAAGGGAAAGUGAGGAAAAAGGUUCCUC
UGUCAACUCCUUACAUAGGAACGUCUUUCAUAUGCAUGAACAACCUCACAACCUUGGCUGGAUCUUUAAG
ACUGAAAG**AUUUA**GCCAUACUAUAAACUGCCUCAAAUGGAACUUUGGGC**AUAAAAGAACUUUUUUUUUAU**
GCUUACCAUUUUUUUUUUCCUUUAACAGAUUUGUAUUUAAGAAUUGUUUUAAAAACUCCUGAAUUUCAC
CCAAUUUUCCUUUGUAAAUAUAGCCAUUAAAUGUAAAUAACUUU**AAUAAAAUUUA**UAGUAGGUAUACCUA
CUAUAUAGUAUAAUUUUU**UUAUUUAA**GUACAUUUUCCUUUUUAAAGUUGAUUUUUUUUCUAUUGUUUUUA
GAAAAA**AUAAA**AUACCUUCAAAUAUAUAAUUGAGCCAAAUCUU

>NM_001046074.2 *Bos taurus MYC* proto-oncogene, bHLH transcription factor (*MYC*), mRNA

GACUCGCUGUAGUAAUUCCAGCGAGAGGCAGAGGGAGCGUGCGGGCGGGCCCUCCAGGGUGGAAGAGCCG
AGCUAGCCUAGCGAGCGGAGUCGCGCUCCGGGCGCCCGGGGAAGGGAGAUCCGGAGUCAAAGGGGGGCUU
CUGCGCCUUCAGCCCGGCCGGCCCCCACCCCACCCACCCCGCGGACCCCUGCUAGCGGUCCGCCACCCGC
GCCGCAUUCGCGAAACUUUGCCCACUGGAACUUACAACAUCCGAGCGACACCAAGACUCCCCGGACGCAG
AGAAGCUCUUCUGCCUUUUUGGGGAGACACUUUCCCCCGCCGCUGCCCACGACCCGCGCCUCUGAAAGGC
GCUCCUCGCCGUUUUUUGGACGCUAGAUUUCCUUCGGAUAGUGGAAAUACGGGCUUGCAGCG**AUGCCCCU**
CAACGUCAGCUUCGCCAACAAGAACUAUGACCUCGACUACGAUUCGGUGCAGCCUUAUUUCUACUGCGAC
GAGGAGGAGAACUUCUACCACCAGCAGCAGCAGAGCGAACUGCAGCCGCCGGCGCCCAGCGAGGAUAUCU
GGAAGAAAUUCGAGCUGCUGCCCACCCCGCCCCUGUCCCCUAGCCGCCGCUCCGGGCUCUGCUCGCCGUC
GUACGUCGCGGUCGCCUCCUUCUCGCCCAGGGGAGACGACGACGGCGGCGGCGGCAGCUUCUCCUCAGCG
GACCAGUUGGAGAUGGUGACCGAGCUACUAGGAGGCGACAUGGUGAACCAGAGCUUCAUCUGCGACCCCG
ACGAUGAGACCCUCAUCAAAAACAUCAUCAUCCAGGACUGUAUGUGGAGCGGCUUCUCGGCCGCCGCCAA
GCUCGUCUCGGAGAAGCUGGCCUCUUACCAGGCUGCGCGCAAAGACGGCGGCAGCCCGAGCCCCGCCCGC
GGGCACGGCGGCUGCUCCACCUCCAGCUUGUACCUGCAGGACCUGAGCGCCGCCGCCUCCGAAUGCAUCG
ACCCCUCGGUGGUCUUCCCCUACCCGCUCAACGACAGCAGCUCGCCCAAGCCCUGCGCUUCCCCGGACUC
CACCGCCUUUUCUCCGUCCUCUGACUCUCUGCUCUCCUCUGCUGAGUCCUCCCCGCGGGCCAGUCCCGAG
CCCCUGGCGCUCCAUGAGGAGACCCCACCCACGACCAGUAGCGACUCUGAGGAAGAACAAGAGGAUGAGG
AAGAAAUUGAUGUUGUUUCUGUGGAAAAGAGGCAGCCCCCUGCCAAAAGGUCAGAAUCGGGGUCACCCUC
UGCCGGCAGCCACAGCAAACCUCCUCACAGCCCGUUAGUCCUAAAGAGAUGCCACGUGUCUACCCAUCAG
CACAAUUACGCAGCGCCCCCCUCCACUAGGAAGGACUAUCCCGCCGCCAAGAGGGCUAAGUUGGACAGUG
GCAGGGUCCUGAAACAGAUCAGCAACAACCGCAAAUGUGCCAGCCCGAGGUCUUCGGACACGGAGGAGAA
UGACAAGAGGCGGACACACAACGUUUUGGAGCGCCAGAGGAGAAACGAGCUGAAACGCAGCUUUUUUGCU
CUUCGUGACCAGAUCCCAGAGUUGGAGAACAAUGAAAAAGCCCCCAAGGUAGUUAUCCUUAAAAAAGCCA
CAGCGUACAUCCUGUCGGUCCAAGCAGAGCAGCAAAAGCUCAAGUCAGAAAUAGACGUGUUGCAGAAGAG
GCGAGAACAGUUGAAACUCAAACUUGAACAGAUACGGAACUCUUGCGCCUAAAUUGACCUAUUGGAGGGA
GGAACUGGACUCCCUCAGGAAAUUCUCAUUUGUUACUAAGGGAAAGUGAGGAAAAAGGUUCCCUCUGUCA
ACUCCUUACAUAGGAACAUCUUUCAUAUGCAUGAACAACCUCACAACCUUGGCUGGAUCUUUAAGACUGA
AAG**AUUUA**GCCAUACUAUAAACUGCCUCAACUUUGGGC**AUAAAAGAACUUUUUUUUUAUGCUUACCAUUU**
UUUUUUUCCUUUAACAGAUUUGUAUUUAAGAAUUGUUUUAAAAACUCCUGAAUUUCACCCAAUUUUCCU
UUGUAAAUAUAGCCAUUAAAUGUAAAUAACUUU**AAUAAAAUUUA**UAGUAGGUAUUCCUACUAUAAUAUAG
GUAUAAUUUUU**UUAUUUAA**GUACAUUUUCCUUUUUAAAGUUGAUUUUUUUUCUAUUGUUUUUAGAAAA**AA**
UAAAAUAUCUUCAAAUAUAUAAUUGAGCCAAAUCUUAAAAA

>XM_005215324.4 Predicted: *Bos taurus MYC* proto-oncogene, bHLH transcription factor (*MYC*), transcript variant X1, mRNA

CUAGCGCCUUUCAUUUUUCCCCAAACCUGGGAGCCCAAGAGUGGUGCAAACCGGCGCCACGGGGCGCAAA
GAGGAUUCGUCUCUUUUCUGAAACCUGGCUGCGGAAUUGGGAACUCCGAGAGAGAGGCGCGGGGGUGGGA
UGGGGGUGUAGACGGACGGGGGUGGUCCCCCGCGGCGCGCACCCGCGGGCGCGCUCCUCCCUUACUCCAG
CUCCGGAACCCGCAGAAAGAUCUCAGGGAUUAGCGCGUACCGCGGGGAGGCUGGGGGAGAGGAACGCUGG
CCCCCGCGGAGAUACGUGAGUUAUUUUCCAAAGGCUGCCCCUUCCCCGGCCUUAGGGAGGCGCCCUUCCA
CCCGGACGUGCAUGGCGCAGCCGUGGGUACACGGUGUAUUCUCAGCGUCGAGGGAUCAGCGGUUCUGCCC
CAGAUGAUUUAAGAACACAGGACAAGUAUGCGGUUUGUCCAACACAGCGCUGCUCCAGAGGAACCAAGCA
GCGCAGAGAGACGGUUUGAGCGGGAGCAAAAGAAAAUGGUAGGCGAGCGCAGUUAAUUCAUGCGGCGCUC
UGACCGUGUUUACACCCGAGAGCUCGACGCAAUGGAGUGCCAGGCUCAAAGAGCUGAAUCUCCUCCCCUC
CUCACCAGCGCCCCUCCCGGGUUCCUAAAGCAGAGAGCGGGGGAGACAGAAAAAAGAUCCUCUCUGGCUU
GCAGCG**AUGCCCCUCAACGUCAGCUUCGCCAACAAGAACUAUGACCUCGACUACGAUUCGGUGCAGCCUU**
AUUUCUACUGCGACGAGGAGGAGAACUUCUACCACCAGCAGCAGCAGAGCGAACUGCAGCCGCCGGCGCC
CAGCGAGGAUAUCUGGAAGAAAUUCGAGCUGCUGCCCACCCCGCCCCUGUCCCCUAGCCGCCGCUCCGGG
CUCUGCUCGCCGUCGUACGUCGCGGUCGCCUCCUUCUCGCCCAGGGGAGACGACGACGGCGGCGGCGGCA
GCUUCUCCUCAGCGGACCAGUUGGAGAUGGUGACCGAGCUACUAGGAGGCGACAUGGUGAACCAGAGCUU
CAUCUGCGACCCCGACGAUGAGACCCUCAUCAAAAACAUCAUCAUCCAGGACUGUAUGUGGAGCGGCUUC
UCGGCCGCCGCCAAGCUCGUCUCGGAGAAGCUGGCCUCUUACCAGGCUGCGCGCAAAGACGGCGGCAGCC
CGAGCCCCGCCCGCGGGCACGGCGGCUGCUCCACCUCCAGCUUGUACCUGCAGGACCUGAGCGCCGCCGC
CUCCGAAUGCAUCGACCCCUCGGUGGUCUUCCCCUACCCGCUCAACGACAGCAGCUCGCCCAAGCCCUGC
GCUUCCCCGGACUCCACCGCCUUUUCUCCGUCCUCUGACUCUCUGCUCUCCUCUGCUGAGUCCUCCCCGC
GGGCCAGUCCCGAGCCCCUGGCGCUCCAUGAGGAGACCCCACCCACGACCAGUAGCGACUCUGAGGAAGA
ACAAGAGGAUGAGGAAGAAAUUGAUGUUGUUUCUGUGGAAAAGAGGCAGCCCCCUGCCAAAAGGUCAGAA
UCGGGGUCACCCUCUGCCGGCAGCCACAGCAAACCUCCUCACAGCCCGUUAGUCCUAAAGAGAUGCCACG
UGUCUACCCAUCAGCACAAUUACGCAGCGCCCCCCUCCACUAGGAAGGACUAUCCCGCCGCCAAGAGGGC
UAAGUUGGACAGUGGCAGGGUCCUGAAACAGAUCAGCAACAACCGCAAAUGUGCCAGCCCGAGGUCUUCG
GACACGGAGGAGAAUGACAAGAGGCGGACACACAACGUUUUGGAGCGCCAGAGGAGAAACGAGCUGAAAC
GCAGCUUUUUUGCUCUUCGUGACCAGAUCCCAGAGUUGGAGAACAAUGAAAAAGCCCCCAAGGUAGUUAU
CCUUAAAAAAGCCACAGCGUACAUCCUGUCGGUCCAAGCAGAGCAGCAAAAGCUCAAGUCAGAAAUAGAC
GUGUUGCAGAAGAGGCGAGAACAGUUGAAACUCAAACUUGAACAGAUACGGAACUCUUGCGCCUAAAUUG
ACCUAUUGGAGGGAGGAACUGGACUCCCUCAGGAAAUUCUCAUUUGUUACUAAGGGAAAGUGAGGAAAAA
GGUUCCCUCUGUCAACUCCUUACAUAGGAACAUCUUUCAUAUGCAUGAACAACCUCACAACCUUGGCUGG
AUCUUUAAGACUGAAAG**AUUUA**GCCAUACUAUAAACUGCCUCAACUUUGGGC**AUAAAAGAACUUUUUUUU**
UAUGCUUACCAUUUUUUUUUUCCUUUAACAGAUUUGUAUUUAAGAAUUGUUUUAAAAACUCCUGAAUUU
CACCCAAUUUUCCUUUGUAAAUAUAGCCAUUAAAUGUAAAUAACUUU**AAUAAAAUUUA**UAGUAGGUAUUC
CUACUAUAAUAUAGGUAUAAUUUUU**UUAUUUAA**GUACAUUUUCCUUUUUAAAGUUGAUUUUUUUUCUAUU
GUUUUUAGAAAA**AAUAAA**AUAUCUUCAAAUAUAUAAUUGAGCCAAA

>XM_010811929.3 Predicted: *Bos taurus MYC* proto-oncogene, bHLH transcription factor (*MYC*), transcript variant X2, mRNA

CGCCGCCUCCCCCGGCCUGUGACGGAGGCCAAAGCUGAUUUCGAUUGCUCGGCGGUGGCGGGGCAGACUC
CCGGGCUUCGCGCUCCAGGCUCCCCGCCGGGGAGCAGAAAAGCCCCGCGGCGCUGAGGAGCUGGUUCGCC
AAGCUUGCAGCG**AUGCCCCUCAACGUCAGCUUCGCCAACAAGAACUAUGACCUCGACUACGAUUCGGUGC**
AGCCUUAUUUCUACUGCGACGAGGAGGAGAACUUCUACCACCAGCAGCAGCAGAGCGAACUGCAGCCGCC
GGCGCCCAGCGAGGAUAUCUGGAAGAAAUUCGAGCUGCUGCCCACCCCGCCCCUGUCCCCUAGCCGCCGC
UCCGGGCUCUGCUCGCCGUCGUACGUCGCGGUCGCCUCCUUCUCGCCCAGGGGAGACGACGACGGCGGCG
GCGGCAGCUUCUCCUCAGCGGACCAGUUGGAGAUGGUGACCGAGCUACUAGGAGGCGACAUGGUGAACCA
GAGCUUCAUCUGCGACCCCGACGAUGAGACCCUCAUCAAAAACAUCAUCAUCCAGGACUGUAUGUGGAGC
GGCUUCUCGGCCGCCGCCAAGCUCGUCUCGGAGAAGCUGGCCUCUUACCAGGCUGCGCAAAGACGGCG
GCAGCCCGAGCCCCGCCCGCGGGCACGGCGGCUGCUCCACCUCCAGCUUGUACCUGCAGGACCUGAGCGC
CGCCGCCUCCGAAUGCAUCGACCCCUCGGUGGUCUUCCCCUACCCGCUCAACGACAGCAGCUCGCCCAAG
CCCUGCGCUUCCCCGGACUCCACCGCCUUUUCUCCGUCCUCUGACUCUCUGCUCUCCUCUGCUGAGUCCU
CCCCGCGGGCCAGUCCCGAGCCCCUGGCGCUCCAUGAGGAGACCCCACCCACGACCAGUAGCGACUCUGA
GGAAGAACAAGAGGAUGAGGAAGAAAUUGAUGUUGUUUCUGUGGAAAAGAGGCAGCCCCCUGCCAAAAGG
UCAGAAUCGGGGUCACCCUCUGCCGGCAGCCACAGCAAACCUCCUCACAGCCCGUUAGUCCUAAAGAGAU
GCCACGUGUCUACCCAUCAGCACAAUUACGCAGCGCCCCCCUCCACUAGGAAGGACUAUCCCGCCGCCAA
GAGGGCUAAGUUGGACAGUGGCAGGGUCCUGAAACAGAUCAGCAACAACCGCAAAUGUGCCAGCCCGAGG
UCUUCGGACACGGAGGAGAAUGACAAGAGGCGGACACACAACGUUUUGGAGCGCCAGAGGAGAAACGAGC
UGAAACGCAGCUUUUUUGCUCUUCGUGACCAGAUCCCAGAGUUGGAGAACAAUGAAAAAGCCCCCAAGGU
AGUUAUCCUUAAAAAAGCCACAGCGUACAUCCUGUCGGUCCAAGCAGAGCAGCAAAAGCUCAAGUCAGAA
AUAGACGUGUUGCAGAAGAGGCGAGAACAGUUGAAACUCAAACUUGAACAGAUACGGAACUCUUGCGCCU
AAAUUGACCUAUUGGAGGGAGGAACUGGACUCCCUCAGGAAAUUCUCAUUUGUUACUAAGGGAAAGUGAG
GAAAAAGGUUCCCUCUGUCAACUCCUUACAUAGGAACAUCUUUCAUAUGCAUGAACAACCUCACAACCUU
GGCUGGAUCUUUAAGACUGAAAG**AUUUA**GCCAUACUAUAAACUGCCUCAACUUUGGGC**AUAAAAGAACUU**
UUUUUUUAUGCUUACCAUUUUUUUUUUCCUUUAACAGAUUUGUAUUUAAGAAUUGUUUUAAAAACUCCU
GAAUUUCACCCAAUUUUCCUUUGUAAAUAUAGCCAUUAAAUGUAAAUAACUUU**AAUAAAAUUUA**UAGUAG
GUAUUCCUACUAUAAUAUAGGUAUAAUUUUU**UUAUUUAA**GUACAUUUUCCUUUUUAAAGUUGAUUUUUUU
UCUAUUGUUUUUAGAAAA**AAUAAA**AUAUCUUCAAAUAUAUAAUUGAGCCAAA

Supplementary Fig. 5. Alignment of *C-MYC* reference protein orthologs from *Mus musculus* (NP_001170823.1), *Ovis aries* (NP_001009426.1), and *Bos taurus* (NP_001039539.1).

```
CLUSTAL multiple sequence alignment by MUSCLE (3.8)


NP_001170823.1      MPLNVNFTNRNYDLDYDSVQPYFICDEEENFYHQQQQSELQPPAPSEDIWKKFELLPTPP
NP_001009426.1      MPLNVSFANRNYDLDYDSVQPYFYCDEEENFYHQQQQSELQPPAPSEDIWKKFELLPTPP
NP_001039539.1      MPLNVSFANKNYDLDYDSVQPYFYCDEEENFYHQQQQSELQPPAPSEDIWKKFELLPTPP
                    *****.*:*.************* ************************************

NP_001170823.1      LSPSRRSGLCSPSYVAVATSFSPREDDDGGGGNFSTADQLEMMTELLGGDMVNQSFICDP
NP_001009426.1      LSPSRRSGLCSPSYVAVA-SFSPRGDDDGGGGSFSSADRLEMVTELLGGDMVNQSFICDP
NP_001039539.1      LSPSRRSGLCSPSYVAVA-SFSPRGDDDGGGGSFSSADQLEMVTELLGGDMVNQSFICDP
                    ****************** ***** *******.**:**.***:*****************

NP_001170823.1      DDETFIKNIIIQDCMWSGFSAAAKLVSEKLASYQAARKDSTSLSPARGHSVCSTSSLYLQ
NP_001009426.1      DDETLIKNIIIQDCMWSGFSAAAKLVSEKLASYQAARKDGGSPSPARGHGGCSTSSLYLQ
NP_001039539.1      DDETLIKNIIIQDCMWSGFSAAAKLVSEKLASYQAARKDGGSPSPARGHGGCSTSSLYLQ
                    ****:**********************************. * ******. *********

NP_001170823.1      DLTAAASECIDPSVVFPYPLNDSSSPKSCTSSDSTAFSPSSDSLLSS-ESSPRASPEPLV
NP_001009426.1      DLSAAASECIDPSVVFPYPLNDSSSPKPCASPDSTAFSPSSDSLLSSAESSPRASPEPLA
NP_001039539.1      DLSAAASECIDPSVVFPYPLNDSSSPKPCASPDSTAFSPSSDSLLSSAESSPRASPEPLA
                    **:************************.*:*.*************** ***********.

NP_001170823.1      LHEETPPTTSSDSEEEQEDEEEIDVVSVEKRQTPAKRSESGSSPSRGHSKPPHSPLVLKR
NP_001009426.1      LHEETPPTTSSDSEEEQEDEEEIDVVSVEKRQPPAKRSESGSPSAGSHSKPPHSPLVLKR
NP_001039539.1      LHEETPPTTSSDSEEEQEDEEEIDVVSVEKRQPPAKRSESGSPSAGSHSKPPHSPLVLKR
                    ********************************.*********..: .*************

NP_001170823.1      CHVSTHQHNYAAPPSTRKDYPAAKRAKLDSGRVLKQISNNRKCSSPRSSDTEENDKRRTH
NP_001009426.1      CHVSTHQHNYAAPPSTRKDYPAAKRAKLDSGRVLKQISNNRKCASPRSSDTEENDKRRTH
NP_001039539.1      CHVSTHQHNYAAPPSTRKDYPAAKRAKLDSGRVLKQISNNRKCASPRSSDTEENDKRRTH
                    *******************************************:****************

NP_001170823.1      NVLERQRRNELKRSFFALRDQIPELENNEKAPKVVILKKATAYILSIQADEHKLTSEKDL
NP_001009426.1      NVLERQRRNELKRSFFALRDQIPELENNEKAPKVVILKKATAYILSVQAEEQKLISEKDV
NP_001039539.1      NVLERQRRNELKRSFFALRDQIPELENNEKAPKVVILKKATAYILSVQAEQQKLKSEIDV
                    **********************************************:**:::** ** *:

NP_001170823.1      LRKRREQLKHKLEQLRNSGA
NP_001009426.1      LRKRREQLKLKLEQIRNSCA
NP_001039539.1      LQKRREQLKLKLEQIRNSCA
                    *.******* ****:*** *
```

Supplementary Fig. 6. Analysis of protein sequence conservation in predicted post-translational modification (PTM) of the *Homo sapiens* *C-MYC* protein. The predicted PTM in the human *C-MYC* by the PhosphoSitePlus* were highlighted (yellow). The protein sequence variation across species was highlighted in red. The transactivation domain (green) and the DNA binding domain (blue) were also highlighted.

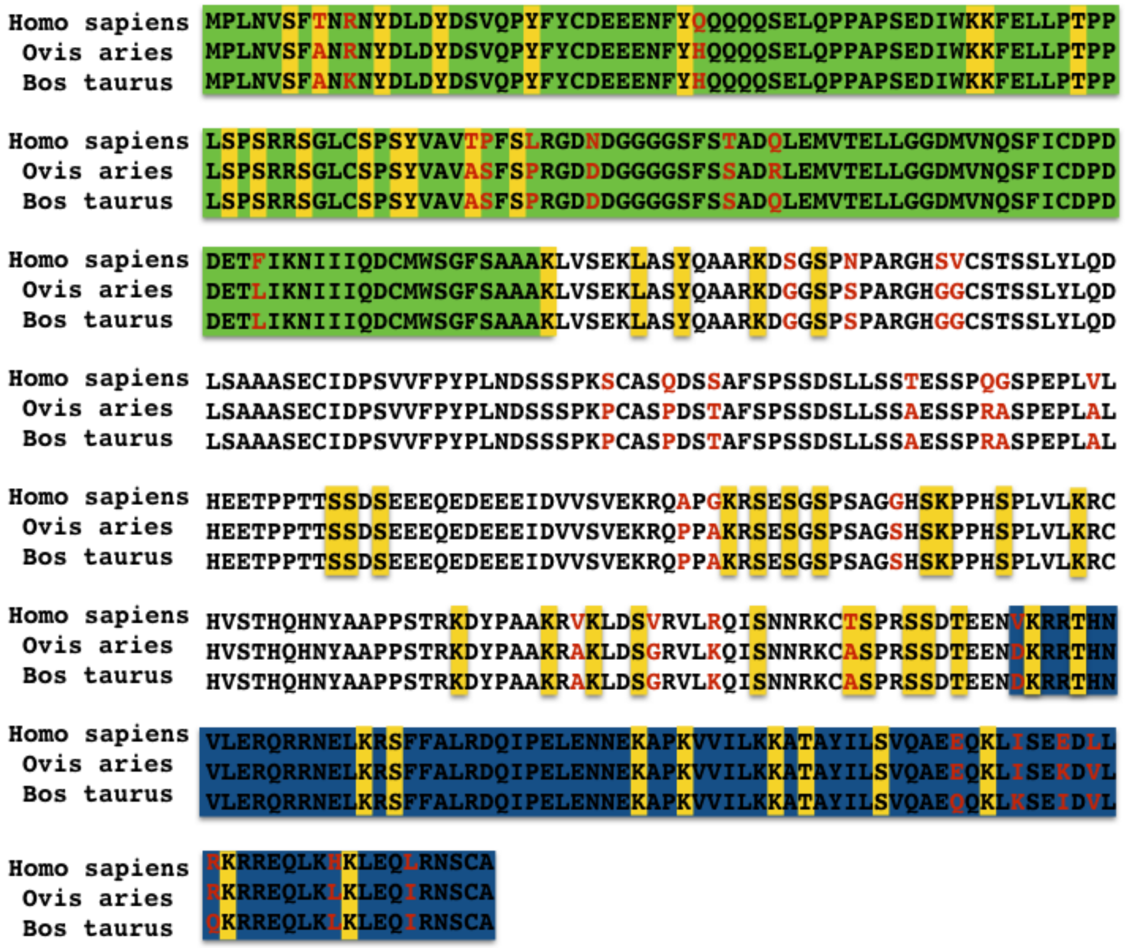

*phosphositeplus.org (September 2019)

Supplementary Table 1. Raw Cq data generated in this study and used for calculation of relative expression of *CDK9*, *C-MYC*, and *C-MYC*-regulators.

	Ovis aries									*Bos taurus*								
**Gene**	**Biological Replicate 1**			**Biological Replicate 2**			**Biological Replicate 3**			**Biological Replicate 1**			**Biological Replicate 2**			**Biological Replicate 3**		
CMYC	20.53	20.10	20.62	22.59	22.18	22.50	19.92	20.13	19.67	21.25	20.68	20.75	22.81	22.78	22.59	24.16	23.74	23.56
CDK9	19.37	18.94	18.60	20.45	21.09	20.91	18.47	19.21	19.38	20.58	20.73	20.69	22.83	22.64	22.47	22.59	22.29	22.44
ATP1A1	16.79	16.86	17.54	19.21	18.53	18.52	16.21	15.63	16.10	18.65	18.78	18.20	21.95	20.86	21.37	21.47	21.49	21.12
RPL19	13.29	13.97	13.81	14.60	14.85	15.14	12.12	12.64	12.64	14.31	14.55	14.66	16.31	16.67	15.90	17.59	16.78	16.82
UBB	15.65	15.33	16.31	17.25	17.43	17.22	14.86	15.11	14.94	16.40	16.05	15.90	18.59	18.47	18.29	19.11	19.05	18.97
RONIN	20.11	19.73	18.87	21.76	21.84	21.83	19.07	20.93	20.78	21.69	21.35	21.12	23.69	23.33	23.74	23.42	23.87	23.38
RXRB	22.05	21.17	21.05	23.65	24.09	23.75	22.15	21.79	21.57	23.47	23.75	23.55	25.93	26.77	25.58	25.53	25.79	25.33
ATP1A1	17.36	16.79	17.51	18.29	18.76	18.30	15.84	15.62	15.75	18.80	18.32	18.24	21.00	21.10	21.45	21.40	21.28	21.71
RPL19	13.83	13.19	13.06	14.78	14.53	14.48	11.92	12.41	12.40	14.47	14.47	14.45	15.88	16.26	16.79	17.64	16.80	16.89
UBB	15.74	15.49	15.93	17.06	16.88	17.44	14.74	14.70	14.62	16.23	16.67	16.89	18.05	18.24	18.29	19.06	19.29	18.23
TBX3	25.64	26.69	25.31	30.17	31.42	28.92	25.18	25.89	25.06	26.69	26.02	26.20	29.26	29.36	30.04	28.27	30.59	29.83
TCF3	23.5	21.80	20.44	23.04	22.87	23.86	20.82	20.48	20.91	22.90	22.55	22.12	26.38	26.23	26.15	25.27	27.20	25.85
ATP1A1	15.69	16.17	16.12	17.62	17.31	17.10	15.53	14.56	14.96	17.46	17.51	16.87	20.81	20.57	20.70	20.53	21.78	20.59
RPL19	12.65	12.33	12.27	14.24	13.51	13.83	10.71	10.68	12.34	13.87	14.00	13.95	15.86	16.08	15.86	16.82	17.00	16.29
UBB	14.38	13.79	15.61	16.62	16.13	17.06	13.19	13.32	14.02	15.70	15.22	14.76	18.14	17.58	18.34	18.48	18.76	18.49

Supplementary Table 2. Relative expression(expression fold of the relative expression of the *B. taurus* relative to the *O. aries* ortholog) using REST version 2.0.13.

Gene	Type	Reaction E	Expressior	Std. Error	95% C.I.	P(H1)	Result
TBX3	TRG	1	3,121	1,014 - 8,529	0,603 - 27,474	0,003	UP
TCF3	TRG	1	0,987	0,510 - 1,925	0,211 - 4,879	0,962	
ATP1A1	REF	1	0,68				
UBB	REF	1	1,517				
RPL19	REF	1	0,969				
TBX3 is UP-regulated in sample group by 3,121 (S.E. range is 1,014 - 8,529).							
TBX3 sample group is different to control group. P(H1)=0,003							
TCF3 sample group is not different to control group. P(H1)=0,962							
Non-Normalised Results							
Gene	Type	Reaction E	Expressior	Std. Error	95% C.I.	P(H1)	Result
TBX3	TRG	1	0,397	0,053 - 3,213	0,026 - 26,538	0,22	
TCF3	TRG	1	0,126	0,024 - 0,652	0,012 - 2,479	0,004	DOWN
ATP1A1	REF	1	0,087	0,020 - 0,399	0,013 - 1,173	0,002	DOWN
UBB	REF	1	0,193	0,039 - 0,717	0,025 - 3,580	0,008	DOWN
RPL19	REF	1	0,123	0,039 - 0,345	0,014 - 1,181	0,002	DOWN
Gene	Type	Reaction E	Expressior	Std. Error	95% C.I.	P(H1)	Result
TBX3	TRG	1	3,073	1,207 - 8,614	0,545 - 30,169	0,004	UP
TCF3	TRG	1	0,972	0,561 - 1,587	0,199 - 4,469	0,916	
ATP1A1	REF	1	0,67				
UBB	REF	1	1,493				
TBX3 is UP-regulated in sample group by 3,073 (S.E. range is 1,207 - 8,614).							
TBX3 sample group is different to control group. P(H1)=0,004							
TCF3 sample group is not different to control group. P(H1)=0,916							
Non-Normalised Results							
Gene	Type	Reaction E	Expressior	Std. Error	95% C.I.	P(H1)	Result
TBX3	TRG	1	0,397	0,053 - 3,213	0,026 - 26,538	0,193	
TCF3	TRG	1	0,126	0,024 - 0,652	0,012 - 2,479	0,003	DOWN
ATP1A1	REF	1	0,087	0,020 - 0,399	0,013 - 1,173	0,001	DOWN
UBB	REF	1	0,193	0,039 - 0,717	0,025 - 3,580	0,01	DOWN
Gene	Type	Reaction E	Expressior	Std. Error	95% C.I.	P(H1)	Result
TBX3	TRG	1	3,844	1,247 - 11,44	0,818 - 37,271	0	UP
TCF3	TRG	1	1,216	0,663 - 2,282	0,324 - 5,796	0,434	
ATP1A1	REF	1	0,838				
RPL19	REF	1	1,193				
TBX3 is UP-regulated in sample group by 3,844 (S.E. range is 1,247 - 11,445).							
TBX3 sample group is different to control group. P(H1)=0,000							
TCF3 sample group is not different to control group. P(H1)=0,434							
Non-Normalised Results							
Gene	Type	Reaction E	Expressior	Std. Error	95% C.I.	P(H1)	Result
TBX3	TRG	1	0,397	0,053 - 3,213	0,026 - 26,538	0,187	
TCF3	TRG	1	0,126	0,024 - 0,652	0,012 - 2,479	0,004	DOWN
ATP1A1	REF	1	0,087	0,020 - 0,399	0,013 - 1,173	0,001	DOWN
RPL19	REF	1	0,123	0,039 - 0,345	0,014 - 1,181	0,001	DOWN

Relative Expression Results							
Gene	Type	Reaction E	Expression	Std. Error	95% C.I.	P(H1)	Result
CMYC	TRG	1	1,829	1,381 - 2,666	0,961 - 3,204	0	UP
CDK9	TRG	1	1,087	0,682 - 1,829	0,375 - 2,962	0,644	
ATP1A1	REF	1	0,602				
RPL19	REF	1	1,113				
UBB	REF	1	1,491				
CMYC is UP-regulated in sample group by 1,829 (S.E. range is 1,381 - 2,666).							
CMYC sample group is different to control group. P(H1)=0,000							
CDK9 sample group is not different to control group. P(H1)=0,644							
Non-Normalised Results							
Gene	Type	Reaction E	Expression	Std. Error	95% C.I.	P(H1)	Result
CMYC	TRG	1	0,338	0,092 - 0,905	0,060 - 3,531	0,013	DOWN
CDK9	TRG	1	0,201	0,077 - 0,401	0,056 - 1,283	0	DOWN
ATP1A1	REF	1	0,111	0,028 - 0,382	0,017 - 1,347	0	DOWN
RPL19	REF	1	0,206	0,061 - 0,605	0,032 - 1,454	0,001	DOWN
UBB	REF	1	0,276	0,080 - 0,781	0,056 - 2,549	0,004	DOWN
Relative Expression Results							
Parameter	Value						
Gene	Type	Reaction E	Expression	Std. Error	95% C.I.	P(H1)	Result
CMYC	TRG	1	2,234	1,591 - 3,293	1,057 - 4,112	0	UP
CDK9	TRG	1	1,327	0,760 - 2,315	0,454 - 3,784	0,15	
ATP1A1	REF	1	0,736				
RPL19	REF	1	1,359				
CMYC is UP-regulated in sample group by 2,234 (S.E. range is 1,591 - 3,293).							
CMYC sample group is different to control group. P(H1)=0,000							
CDK9 sample group is not different to control group. P(H1)=0,150							
Non-Normalised Results							
Gene	Type	Reaction E	Expression	Std. Error	95% C.I.	P(H1)	Result
CMYC	TRG	1	0,338	0,092 - 0,905	0,060 - 3,531	0,014	DOWN
CDK9	TRG	1	0,201	0,077 - 0,401	0,056 - 1,283	0,001	DOWN
ATP1A1	REF	1	0,111	0,028 - 0,382	0,017 - 1,347	0,001	DOWN
RPL19	REF	1	0,206	0,061 - 0,605	0,032 - 1,454	0,001	DOWN
Relative Expression Results							
Gene	Type	Reaction E	Expression	Std. Error	95% C.I.	P(H1)	Result
CMYC	TRG	1	1,93	1,363 - 2,878	0,997 - 4,000	0	UP
CDK9	TRG	1	1,146	0,639 - 1,925	0,374 - 2,949	0,514	
ATP1A1	REF	1	0,636				
UBB	REF	1	1,573				
CMYC is UP-regulated in sample group by 1,930 (S.E. range is 1,363 - 2,878).							
CMYC sample group is different to control group. P(H1)=0,000							
CDK9 sample group is not different to control group. P(H1)=0,514							
Non-Normalised Results							
Gene	Type	Reaction E	Expression	Std. Error	95% C.I.	P(H1)	Result
CMYC	TRG	1	0,338	0,092 - 0,905	0,060 - 3,531	0,02	DOWN
CDK9	TRG	1	0,201	0,077 - 0,401	0,056 - 1,283	0,001	DOWN
ATP1A1	REF	1	0,111	0,028 - 0,382	0,017 - 1,347	0,001	DOWN
UBB	REF	1	0,276	0,080 - 0,781	0,056 - 2,549	0,008	DOWN

Relative Expression Results							
Gene	Type	Reaction Ef	Expression	Std. Error	95% C.I.	P(H1)	Result
RONIN	TRG	1	1,243	0,700 - 3,108	0,347 - 4,757	0,407	
RXRB	TRG	1	0,93	0,455 - 1,810	0,238 - 2,609	0,75	
ATP1A1	REF	1	0,65				
UBB	REF	1	1,486				
RPL19	REF	1	1,035				
RONIN sample group is not different to control group. P(H1)=0,407							
RXRB sample group is not different to control group. P(H1)=0,750							
Non-Normalised Results							
Gene	Type	Reaction Ef	Expression	Std. Error	95% C.I.	P(H1)	Result
RONIN	TRG	1	0,204	0,061 - 0,614	0,035 - 1,558	0,003	DOWN
RXRB	TRG	1	0,152	0,048 - 0,370	0,027 - 1,266	0,002	DOWN
ATP1A1	REF	1	0,106	0,024 - 0,438	0,017 - 1,042	0,001	DOWN
UBB	REF	1	0,243	0,085 - 0,599	0,043 - 1,705	0,002	DOWN
RPL19	REF	1	0,169	0,048 - 0,431	0,027 - 1,240	0,002	DOWN
Relative Expression Results							
Gene	Type	Reaction Ef	Expression	Std. Error	95% C.I.	P(H1)	Result
RONIN	TRG	1	1,264	0,736 - 3,315	0,304 - 5,134	0,362	
RXRB	TRG	1	0,946	0,481 - 1,849	0,238 - 2,558	0,804	
ATP1A1	REF	1	0,662				
RPL19	REF	1	1,512				
Interpretation							
RONIN sample group is not different to control group. P(H1)=0,362							
RXRB sample group is not different to control group. P(H1)=0,804							
Non-Normalised Results							
Gene	Type	Reaction Ef	Expression	Std. Error	95% C.I.	P(H1)	Result
RONIN	TRG	1	0,204	0,061 - 0,614	0,035 - 1,558	0,001	DOWN
RXRB	TRG	1	0,152	0,048 - 0,370	0,027 - 1,266	0,001	DOWN
ATP1A1	REF	1	0,106	0,024 - 0,438	0,017 - 1,042	0	DOWN
RPL19	REF	1	0,243	0,085 - 0,599	0,043 - 1,705	0,002	DOWN
Relative Expression Results							
Parameter	Value						
Iterations	2000						
Gene	Type	Reaction Ef	Expression	Std. Error	95% C.I.	P(H1)	Result
RONIN	TRG	1	1,264	0,736 - 3,315	0,304 - 5,134	0,37	
RXRB	TRG	1	0,946	0,481 - 1,849	0,238 - 2,558	0,802	
ATP1A1	REF	1	0,662				
UBB	REF	1	1,512				
Interpretation							
RONIN sample group is not different to control group. P(H1)=0,370							
RXRB sample group is not different to control group. P(H1)=0,802							
Non-Normalised Results							
Gene	Type	Reaction Ef	Expression	Std. Error	95% C.I.	P(H1)	Result
RONIN	TRG	1	0,204	0,061 - 0,614	0,035 - 1,558	0,001	DOWN
RXRB	TRG	1	0,152	0,048 - 0,370	0,027 - 1,266	0,001	DOWN
ATP1A1	REF	1	0,106	0,024 - 0,438	0,017 - 1,042	0,001	DOWN
UBB	REF	1	0,243	0,085 - 0,599	0,043 - 1,705	0,002	DOWN
